# Limit-Cycle Proliferation Under Parametric Delayed Feedback in a Conductance-Based Neuron: Bifurcation Landscape, Orbit Catalog, and Capacity Analysis

**DOI:** 10.3390/e28060678

**Published:** 2026-06-11

**Authors:** Mohammad O. Alhawarat, Ayman J. Alnsour, Mohammed A. F. Al-Husainy, Khalil M. Abdelnaby

**Affiliations:** 1 Faculty of Information Technology, Al-Ahliyya Amman University, Amman 19328, Jordan; a.alnsour@ammanu.edu.jo (A.J.A.); m.alhusainy@ammanu.edu.jo (M.A.F.A.-H.); k.abdelnaby@ammanu.edu.jo (K.M.A.); 2 Systems and Computers Engineering Department, Faculty of Engineering, Al-Azhar University, Nasr City, Cairo 11765, Egypt

**Keywords:** Hodgkin–Huxley model, parametric delayed feedback, limit-cycle multiplicity, orbit-coded memory, bifurcation landscape, autaptic feedback, neuromorphic computing

## Abstract

We show that a single Hodgkin–Huxley (HH) neuron with Pyragas-type delayed feedback control (DFC) can store multiple symbols as stable periodic orbits, where the specific orbit is selected by tuning the DFC gain *K* and time delay τ. Sweeping the (K,τ) parameter plane at fixed bias current $I_{\mathrm{bias}}$ = 10.0 μA/cm^2^ reveals 207 orbit types across 12 topological categories, with inter-spike interval (ISI) means from 5.9 to 56.9 ms. We establish: (i) a write protocol that reliably locks orbits with 13.9 ms median settling time; (ii) a novel Pattern-Oriented Limit-cycle Decoder (POLD) that reads orbits at 100% accuracy from only five observed ISIs (1200 trials across 12 orbits; Wilson 95% CI: 99.7–100%); (iii) a complete single-symbol write–read–erase (W–R–E) cycle with 100% read accuracy, 92% erase verification, and no decay over hold durations up to 50 s; and (iv) a fully validated 12-symbol memory capacity with a read-discriminable upper bound of 67 symbols (11.2× over rate coding; write viability confirmed only for the conservative 12-symbol subset). Reliable orbit addressing needs delay precision of ±2%, which constitutes a write-precision specification and not a fundamental capacity limit. These findings show that parametric delayed feedback is a viable mechanism for limit-cycle-based information storage in conductance-based spiking neurons. The biological interpretation is analogical, not direct: the ±2% delay-precision requirement exceeds what has been demonstrated for biological autaptic variability, and the orbit-coded memory framing is best understood as a computational proof-of-principle aimed at neuromorphic engineering, not as a claim about biological working memory.

## 1. Introduction

A central question in computational neuroscience concerns the computational repertoire of individual neurons. Classically, neurons are assigned a straightforward input–output role: they integrate synaptic currents and report their mean firing rate as an output signal. This rate-coding hypothesis [1,2] has been enormously productive, but accumulating evidence suggests that single neurons can support far richer computations through their intrinsic dynamics. Precise spike timing carries information beyond what is captured by firing rate [3,4]; individual dendrites perform local, nonlinear computations that effectively make a single cell a multi-layer processing unit [5]; and intrinsic bistability or multistability can give individual neurons the ability to sustain distinct activity states in the absence of ongoing input [6,7]. The present work explores an extreme case of this last capacity: using the dynamical richness of a single Hodgkin–Huxley neuron’s limit-cycle repertoire for multi-symbol information storage. It is important to distinguish the type of memory demonstrated here: this is *location-addressable* (symbolic) memory, where a discrete symbol is written, held, and read back via an explicit address (the parameter pair (K,τ)), analogous to a multi-level memory cell. This differs fundamentally from *content-addressable* (associative) memory, where a noisy or partial input pattern is attracted to the nearest stored pattern (e.g., Hopfield networks [8]). The practical engineering motivation is the design of compact, multi-state neuromorphic memory cells for edge computing hardware, where storing more than two states per neuron-equivalent circuit element directly reduces device count and energy cost [9]. This demand is well-established: Cao et al. [10] review multi-state single-cell memories and note that multi-states in a single cell provide an unconventional in-memory computing platform beyond Von Neumann architecture; Rzeszut et al. [11] demonstrate multi-state MRAM cells for hardware neural computing; and Park et al. [12] implement 100-level single-element RRAM for neuromorphic edge computing. Our work provides an alternative, purely dynamical approach to this challenge.

The working memory problem—how the brain maintains information over seconds without external reinforcement—has traditionally been modeled at the network level. Attractor network models, originating with Hopfield [8] and extended to spiking networks by Amit and Brunel [13] and Wang [14], posit that patterns of activity are held as stable fixed points of recurrent synaptic dynamics. Recent extensions incorporate activity-dependent plasticity and dynamic memory representations [15,16]. These rely on structured connectivity and balance between excitation and inhibition; their capability depends on the size of the network. The single neuron bistability models [6,7,17] offer a complementary approach to maintain bistable regimes in the absence of network structure through the dynamics of intrinsic conductance parameters. This involves models involving persistent Na^+^ conductance [18], K^+^ slow conductances [19], and facilitated synapses due to astrocytes [20]. Our strategy stands out in that it takes advantage of the inherent multistability property of a delay differential equation (DDE) model where the neuron’s previous output serves as feedback to create a high-dimensional effective state space.

Time-lagged self-feedback occurs physiologically in neurons due to autaptic synapses (synapses created between an axon terminal from the same neuron and the dendrites of that neuron). Initially discovered anatomically by van der Loos and Glaser [21], autapses have been observed all across the neocortex [22] and hippocampal circuits [23] to date. Autapses contribute to neurobiological phenomena such as modulation of firing precision [24], gain control [25], oscillations generation [26], and coherence resonance in autaptically regulated networks [27,28,29]. Crucially, autaptic feedback directly shapes burst firing patterns with varied ISI sequences: Yin et al. [30] documented excitatory autapses in both rodent and human layer-5 neocortical pyramidal cells that enhance burst firing and produce postsynaptic responses approximately five-fold larger than recurrent synapses; Deleuze et al. [31] showed that autaptic transmission is the most powerful inhibitory input to neocortical PV interneurons and directly modulates their gamma-frequency burst coupling; and Wang et al. [32] showed that time-delayed autaptic feedback controls mode transitions between distinct burst firing patterns. These results establish that the autaptic self-feedback mechanism that we model is biologically documented and directly relevant to the burst-pattern diversity demonstrated in this study. Candidate biological mechanisms for tuning these parameters exist: *K* can be modulated through activity-dependent plasticity of autaptic conductance [24,26], and τ can be adjusted through myelination state and oligodendrocyte-mediated axonal plasticity [33,34]; these mechanisms are discussed quantitatively in Section 5.3. Crucially, both excitatory and inhibitory autapses have been documented. The current work limits K≥0 (excitatory self-feedback); inhibitory autapses have been shown capable of providing effective sign change in feedback due to circuit polarity inversion, but their exploration is deferred to future work. We emphasize that the autaptic analogy serves as a computational motivation for the parameter regime, not a direct biological claim; the ±2% delay-precision requirement identified in Section 4.3 exceeds demonstrated biological autaptic variability, and the contribution is best positioned as a neuromorphic engineering proof-of-principle (Section 5.3).

Pyragas [35] introduced time-delayed feedback control (DFC) as a method for stabilizing unstable periodic orbits (UPOs) embedded in chaotic attractors. The control signal $I_{\mathrm{ctrl}}\left(t\right)=K\left[V\left(t-\tau \right)-V\left(t\right)\right]$ vanishes exactly when V(t) is periodic with period τ, so the target orbit becomes a fixed point of the controlled dynamics while remaining physically equivalent to the uncontrolled orbit. Early applications focused on optics [36] and semiconductor lasers [37]. He et al. [38] verified selective UPO stabilization in a chaotic neural network; lately, DFC tested in vitro on biological neuronal populations for desynchronization of pathological oscillations [39]. The foundational concept of orbit-coded memory was introduced by Crook, Goh, and Hawarat [40,41] using the nonlinear dynamic state (NDS) neuron—a Rössler-based discrete-time map. In the NDS approach, the system operates at a single fixed parameter point within a chaotic attractor; DFC is applied as an active control perturbation that continuously forces the trajectory to stay near one specific UPO embedded in that attractor. Memory is encoded as a choice of which UPO to stabilize, and the control signal must remain active throughout memory maintenance—removing it allows the trajectory to escape back into chaos. Subsequent analyses [42,43,44,45,46] characterized the NDS model’s dynamics and memory properties in detail.

The present work is methodologically distinct from the NDS approach in every essential respect. We do not operate within a chaotic attractor, we do not stabilize UPOs, and we do not require any ongoing adjustment of control parameters during memory maintenance. Instead, we exploit the fact that changing (K,τ) moves the HH–DFC system to a different region of its bifurcation landscape, where a different stable periodic orbit is the natural attractor. Once the neuron locks onto this orbit, the DFC term K[V(t−τ)−V(t)] vanishes exactly for orbits whose period matches τ; for higher-order burst orbits whose fundamental period does not equal τ, the DFC term participates actively in the dynamics (see Section 5.1). In both cases, the memory is preserved without having to stabilize it actively. The write process involves switching the parameters and does not involve orbit stabilization. Therefore, the memory configuration is that of a stable attractor and not that of an unstable controlled attractor. There is more to this difference than meets the eye since it affects the system’s capacity, robustness, and maintenance costs.

The challenge of extending orbit-coded memory into biophysically realistic conductance-based neurons remains open. This is fundamental: the Hodgkin–Huxley model chaotic regime, defined by Guckenheimer and Oliva [47], allows for only two to four ISI distinctive clusters—far fewer than the around 15 orbits needed for useful memory capacity. Aihara, Matsumoto, and Ikegaya [48] demonstrated that periodic and chaotic responses coexist in the forced HH oscillator, but the diversity of periodic states reachable via chaos control alone is limited by the spiking mechanism, which constrains the action potential waveform. A key insight motivating our work is that this scarcity pertains specifically to the chaotic regime; the stable periodic regime of the HH–DFC system is, as we shall show, vastly richer.

Why such richness? Yanchuk and colleagues [49,50] showed that delay-coupled neural oscillators support dense families of coexisting stable periodic orbits, born through Hopf bifurcations as the delay grows. Kantner, Yanchuk, and Schöll [51] further showed that coupling delay greatly increases both the number and stability of periodic solutions in neural networks. More recently, photonic neuron models with delayed self-feedback [52,53] showed that such systems can hold multiple coexisting temporal localized states, each one a different memory symbol, and that brief perturbations can switch between them. Those photonic implementations used simplified FitzHugh–Nagumo dynamics and stored binary patterns. The specific architecture of a single autaptic oscillatory neuron as a memory element has since been demonstrated in hardware: Romeira et al. [54] implemented a photonic regenerative memory using a FitzHugh–Nagumo oscillator with delayed self-feedback, demonstrating proof-of-concept optical buffer memory; Romeira, Figueiredo, and Javaloyes [55] reviewed the broader program covering spike-based data encoding, storage, and signal regeneration in autaptic artificial neurons; and Stoliar et al. [56] implemented the architecture in solid-state electronics, demonstrating a tunable dynamic memory with graded persistent activity. Our contribution extends this established architecture from one to two addressable states to 12 write-validated states with a complete write–read–erase protocol and systematic capacity characterization. Here, we show analogous functionality in the full Hodgkin–Huxley conductance model, reaching a read-discriminable capacity of 67 symbols (pending write-viability confirmation; full W–R–E viability confirmed for 12 symbols) with a complete W–R–E protocol and systematic capacity characterization.

To place the contribution in context, the uncontrolled HH neuron (K=0) supports only six distinguishable tonic firing states across its full physiological range (Section 4.5). Adding the DFC term with two control parameters (K,τ) expands this to 207 distinct orbit types across 12 topological categories—a 34-fold increase in addressable states within the same physical substrate, arising from a non-trivial Hopf bifurcation proliferation mechanism [49,50] that does not occur in the uncontrolled model.

What sets our approach apart from earlier DFC-based neural memory is one key point: instead of stabilizing UPOs inside a fixed chaotic attractor, we use (K,τ) as an address that moves the HH–DFC system to a different part of its bifurcation landscape, where a different stable periodic attractor lives. This shift—from chaos-control memory to bifurcation-directed memory—sidesteps the topological constraints of the chaotic regime and opens up hundreds of distinct orbit types in 12 qualitative categories. The mechanism is also conceptually related to reservoir computing [57,58] in that the neuron’s internal dynamics (here, a delay differential equation) generate a rich representational space; however, unlike reservoir computing, our system stores information in a stable attractor rather than in a transient trajectory, enabling retention without ongoing input.

A note on terminology: Crook et al. [40,41] coined orbit-coded memory in the NDS context. We keep the term because the idea behind encoding information in distinct periodic orbits is the same—although our mechanism is fundamentally different (Section 5.1). Memory is the computational property of persistent, switchable, distinguishable dynamical states; it is not biological memory in the cognitive or synaptic sense used herein. This work is motivated by biological reasons rather than mechanistic claims; the autaptic analogy (Section 5.3) is computationally motivated toward neuromorphic engineering applications.

A note on cognitive neuroscience analogies: Throughout this paper, references to established cognitive science benchmarks (Miller’s 7 ± 2 working memory span, Baddeley’s maintenance window, and behavioral encoding timescales) serve exclusively as computational reference benchmarks for contextualizing simulation parameters (gate thresholds, hold durations, settling times). They do not constitute mechanistic claims: this study provides no evidence of cortical implementation, does not incorporate synaptic plasticity, and is not validated at the network or population level. The orbit-coded memory framing is a computational proof-of-principle aimed at neuromorphic engineering.

This paper makes the following contributions. (C1) We catalog 207 distinct stable periodic orbit types in the HH–DFC system from a dense 10,100-point sweep of the (K,τ) plane. (C2) We design and validate a complete W–R–E memory cycle achieving 100% read accuracy and 92% erase verification across 12 representative orbits spanning all topological categories, with no decay observed over hold durations up to 50 s. (C3) We introduce the Pattern-Oriented Limit-cycle Decoder (POLD), a lightweight classifier that achieves 100% read accuracy using only five observed ISIs, with no training data required. (C4) We establish a read-discriminable capacity of 67 symbols via a full pairwise confusion matrix and greedy subset selection; full write–read–erase viability is confirmed for a conservative 12-symbol library. (C5) We demonstrate an 11.2× read-discriminable capacity advantage over rate coding (K=0, variable $I_{\mathrm{bias}}$) in the same neuron; this figure refers to read-discriminable capacity only, with full W–R–E viability confirmed for the conservative 2.0× advantage (12 vs. 6 symbols). (C6) We provide a fully reproducible, gate-based experimental pipeline (PS0–PS5) with formal pass/fail criteria at each phase.

The rest of this paper is arranged as follows: Section 2 defines the mathematical framework including HH-DFC, orbit library formalism and POLD classifier. Section 3 describes the six-phase experimental pipeline. In Section 4, we show results, whereas Section 5 reports on implications, limitations, and future directions. Finally, Section 6 concludes the paper.

## 2. Mathematical Framework

### 2.1. Hodgkin–Huxley Neuron with Delayed Feedback Control

Consider the standard Hodgkin–Huxley equations [59] augmented with a Pyragas-type DFC term [35]:

(1) $$C_{m}\frac{dV}{dt}=I_{\mathrm{bias}}+K\left[V(t-\tau )-V(t)\right]-{\bar{g}}_{\mathrm{Na}}\;m^{3}h\left(V-E_{\mathrm{Na}}\right)-{\bar{g}}_{K}\;n^{4}\left(V-E_{K}\right)-{\bar{g}}_{L}\left(V-E_{L}\right)$$

where V(t) is the membrane potential (mV), and m,h,n∈[0,1] are the sodium activation, sodium inactivation, and potassium activation gating variables, each satisfying

(2) $$\frac{dx}{dt}=\alpha_{x}\left(V\right)\left(1-x\right)-\beta_{x}\left(V\right)\;x$$

where standard Hodgkin–Huxley rate functions are given in Appendix A. The control parameters are: K≥0 (DFC gain, mS/cm^2^), τ>0 (time delay, ms), and $I_{\mathrm{bias}}$ (constant injected current, μA/cm^2^). We use the *V*-shifted convention with resting potential $V_{\mathrm{rest}}=0$ mV and standard squid giant axon parameters: $C_{m}=1$ μF/cm^2^; ${\bar{g}}_{\mathrm{Na}}=120$, ${\bar{g}}_{K}=36$, ${\bar{g}}_{L}$ = 0.3 mS/cm^2^; $E_{\mathrm{Na}}=115$, $E_{K}=-12$, $E_{L}$ = 10.6 mV. All simulations use $I_{\mathrm{bias}}$ = 10.0 μA/cm^2^ unless stated otherwise. The DFC term models an idealized electrical (gap-junction-type) autaptic coupling. This differs from chemical (spike-mediated) autaptic transmission, which is more common in the biological autapse literature; a chemical autaptic implementation would require additional synaptic kinetics modeling beyond the scope of this study. The standard squid giant axon parameters are retained without optimization: the goal is to characterize the capacity of the HH–DFC architecture in a biologically validated, kinetically complete model before simplification for hardware deployment. Martins, Gurevich, and Javaloyes [60] show that, for related autaptic oscillator systems, the simplified FitzHugh–Nagumo model captures the dynamics only in limiting parameter regimes, and that detailed model kinetics govern the achievable memory capacity. This motivates using the full HH model for capacity analysis; simplification to Morris–Lecar or FitzHugh–Nagumo is identified as the next step in Section 5.6.

The HH–DFC system is a DDE of dimension 4+∞ (the finite-dimensional state plus the delay history). The DFC term adds a perturbation to the membrane current that is proportional to the difference between the current voltage and the voltage one delay period ago. When V(t) is periodic with period τ, this term vanishes exactly, which is the key non-invasive property of Pyragas control [35,61]; for orbits whose period differs from τ, the DFC term remains dynamically active (see Section 5.1). Importantly, the DFC term operates in two distinct regimes: (i) for orbits whose fundamental period matches τ, the term vanishes exactly (the classical Pyragas non-invasive property), with RMS control current below 2% of the bias current; (ii) for higher-order burst orbits whose period differs from τ, the DFC term contributes substantially (RMS control current 15–140% of bias) and functions as an integral shaping force in the modified dynamical landscape rather than a small corrective perturbation. In both regimes, the target orbit is a stable attractor of the full HH–DFC system; quantitative characterization is presented in Section 4.1 and interpreted in Section 5.1. For K=0, Equation (1) reduces to the standard uncontrolled HH model. Floquet multipliers for the full orbit library are not computed here; stability is established empirically via sustained periodic behavior over extended hold durations (up to 50 s). As a representative validation, Floquet multipliers were computed for nine orbits spanning all topological categories (tonic, doublet, triplet, and burst_p4 through burst_p9); results are reported in Table 1.

**Stability convention.** Throughout this paper, ‘stable’ refers to empirically established stability: sustained periodic behavior under the tested simulation conditions (RK4, dt=0.01 ms, hold durations up to 50 s) without decay or regime change. This empirical criterion is applied uniformly across all 207 orbit types. Floquet multiplier computations for nine representative orbits spanning all topological categories (Table 1,) provide partial corroboration of stability but are not the basis of our stability claims, given the computational challenges of Floquet analysis for infinite-dimensional DDE systems; the empirical criterion (sustained periodic behavior over 50 s without decay or regime change) is applied uniformly to all 207 orbit types and constitutes the primary stability validation. All subsequent references to orbit stability carry this qualification unless otherwise noted.

We integrate numerically with fourth-order Runge–Kutta at dt=0.01 ms, which is fine for reproducing spike waveforms given the 0.5–2.0 ms spike width of HH action potentials. The delay buffer is implemented as a circular array of length ⌈τ/dt⌉ and warm-started using the RC3 protocol: the neuron free-runs with K=0 for the first τ ms to fill the buffer with physiological voltage history, eliminating artificial transients from zero-initialized buffers.

### 2.2. Stability and the Orbit Landscape

A periodic orbit O of period *T* has Floquet multipliers $\mu_{i}$—the eigenvalues of the monodromy matrix M=Φ(T,0), with Φ being the fundamental solution matrix of the variational equation [62]. In the DDE (1), *M* is an infinite-dimensional operator in the delay history space. Its spectrum contains one trivial multiplier, μ=1 (phase shift along the orbit), and countably many stability-determining multipliers. An orbit is asymptotically stable (an attractor) if all non-trivial Floquet multipliers fulfill $|\mu_{i}|\;<\;1$. We computed Floquet multipliers for nine representative orbits (as a check on the empirical stability criterion used in this study) covering all major topological categories (tonic, doublet, triplet, and burst_p4 through burst_p9) via variational QR-iteration; results appear in Table 1.

There is a direct theoretical reason for the richness of the HH–DFC orbit landscape. For a scalar DDE with delay τ, the number of coexisting stable periodic orbits grows approximately as $\tau /T_{0}$, where $T_{0}$ is the characteristic oscillation period [49]. For the HH neuron at $I_{\mathrm{bias}}=10.0$ μA/cm^2^, the natural ISI is approximately $T_{0}\approx 10.6$ ms (firing rate ∼94 Hz). For τ=100 ms, we therefore expect O(10) coexisting periodic solutions, and our numerical sweep does find hundreds across the full (K,τ) plane. As τ increases, new periodic orbits are born through Hopf bifurcations at which a pair of complex conjugate Floquet multipliers crosses the unit circle. Yanchuk and Perlikowski [50] showed that these bifurcations accumulate densely as τ→∞, producing an asymptotically unbounded number of coexisting stable orbits—which is exactly what we exploit to build a large orbit catalog. Recent work has further confirmed this mechanism in biophysically realistic settings: Laing and Krauskopf [63] demonstrated analytically that a single theta neuron with delayed self-feedback sustains multiple coexisting periodic solutions with increasing multistability as delay grows, and Wedgwood et al. [64] confirmed experimentally the coexistence of multiple spike patterns in an excitable cell with delayed feedback.

### 2.3. Orbit Library and Memory Formalism

**Definition 1**  (Orbit type)**.** *An orbit type $O_{i}$ is an equivalence class of stable periodic trajectories of Equation (1) characterized by a canonical ISI pattern: the ordered sequence of inter-spike intervals within one fundamental period. Two trajectories belong to the same orbit type if their ISI pattern vectors are within 2 ms Euclidean distance in ISI-mean space. This threshold is motivated by the minimum ISI jitter arising from intrinsic conductance noise and numerical discretization ($\sigma_{ISI}<0.05$ ms for all stable orbits observed).*

**Definition 2**  (Orbit categories)**.** *Orbits are assigned to qualitative categories based on their pattern length p (number of distinct ISIs per period): tonic (p=1), doublet (p=2), triplet (p=3), and burst_pν (p=ν) for ν=4,…,12. Within each category, orbits differ in their mean ISI and in the fine structure of their ISI pattern vector.*

**Definition 3**  (Libraries)**.** The catalog library $L_{cat}$ is the set of all distinct orbit types identified across the full (K,τ) parameter sweep. The addressable library $L_{addr}\subseteq L_{cat}$ is the subset whose members satisfy write viability (lock rate ≥80%) and read viability (pairwise classification accuracy ≥90%). The number of simultaneously unique symbols is considered as the memory capacity ($N^{*}=\left|L_{addr}\right|$). A read-discriminable subset $L_{read}\subseteq L_{cat}$ satisfies the read viability criterion only; its cardinality $N_{read}^{*}=\left|L_{read}\right|$ is the read-discriminable capacity, which serves as an upper bound on $N^{*}$ that becomes exact once write viability is confirmed for all members.

**Capacity terminology.** Throughout this paper, $N^{*}=12$ denotes the *write-validated capacity*: the number of symbols for which the complete write–read–erase cycle has been confirmed. $N_{\mathrm{read}}^{*}=67$ denotes the *read-discriminable capacity*: the number of symbols confirmed to be pairwise distinguishable by POLD, pending write-viability confirmation for the extended set. These two quantities represent fundamentally different notions of capacity and are not interchangeable. Every occurrence of the 67-symbol or 11.2× figures in this paper refers exclusively to read-discriminable capacity.

HH-DFC neurons are thought of as devices for storing memories that can perform three operations:

1. **Write:** Instantaneous switch of DFC parameters from a baseline state (K=0, tonic firing) to target values $\left(K_{i},\tau_{i}\right)$ that cause the neuron to move to orbit $O_{i}$. Locking is achieved when the average of five consecutive ISIs falls within 5% of the target ISI mean and when the CV of these five ISIs is less than 0.05. Settling time is then measured from the parameter switch to the first spike of the locking window.
2. **Read:** Recording the spike train during the read window and classifying the orbit using POLD based on the ISI fingerprint.
3. **Erase:** Changing the DFC parameters back to K=0, resulting in tonic firing. Validation of erasure is done by applying POLD using five ISIs in the post-reset window, where erasure is successful when the classifier gives an orbit that differs from the written symbol.

### 2.4. ISI-Based Orbit Classification: POLD

The Pattern-Oriented Limit-cycle Decoder (POLD) classifies the neuron’s current orbit from an observed sequence of *n* ISIs, denoted $\vec{\iota }=\left(\iota_{1},\ldots ,\iota_{n}\right)$. For each candidate orbit template $O_{j}$ in the library (with mean $\mu_{j}$, standard deviation $\sigma_{j}$, and canonical pattern vector ${\vec{\rho }}_{j}$ of length $p_{j}$), POLD computes

(3) $$S_{j}=0.6\cdot S_{\mathrm{mean}}\left(j\right)+0.4\cdot S_{\mathrm{pattern}}\left(j\right)$$

where the mean score is a Gaussian kernel on the normalized ISI mean distance:

(4) $$S_{\mathrm{mean}}\left(j\right)=\exp\;\left(-0.5\;z_{j}^{2}\right),\;z_{j}=\frac{|\mu_{\mathrm{obs}}-\mu_{j}|}{\max(\sigma_{j},\;0.5)}$$

where $\mu_{\mathrm{obs}}=n^{-1}\sum_{i}\iota_{i}$ is the observed mean ISI over the read window. The pattern score is

(5) $$S_{\mathrm{pattern}}\left(j\right)=\max_{k}\Psi \;\left(\vec{\iota }\left[k:k+p_{j}\right],\;{\vec{\rho }}_{j}\right)$$

where ${\vec{\iota }}_{\mathrm{seg}}=\vec{\iota }\left[k:k+p_{j}\right]$ denotes a length-$p_{j}$ segment of the observed ISI sequence starting at window index *k*, corr(·,·) is the Pearson correlation coefficient (with negative values floored at zero), and $\mathrm{rel}\_\mathrm{err}=\left(1/p_{j}\right)\sum_{i}\left|\iota_{\mathrm{seg},i}-\rho_{j,i}\right|/\left(\rho_{j,i}+0.1\right)$ is the mean element-wise relative error between the observed segment and the template pattern, with a 0.1 ms floor in the denominator to prevent division by zero. The window score is

(6) $$\Psi \;\left({\vec{\iota }}_{\mathrm{seg}},\;{\vec{\rho }}_{j}\right)=0.5\cdot \mathrm{corr}\left({\vec{\iota }}_{\mathrm{seg}},\;{\vec{\rho }}_{j}\right)+0.5\cdot \exp\left(-2\cdot \mathrm{rel}\_\mathrm{err}\right)$$

where correlation strength is combined with a relative error penalty. The prediction is $\hat{𝚤}=\arg\max_{j}S_{j}$. POLD requires no training data: templates are built from the mean ISI and mean pattern vector of five long simulation runs per orbit. The classifier operates in O(nL) time per observation, where *L* is the library size, making it suitable for real-time implementation. This ratio of 60/40 between mean-score and pattern-score was selected empirically. A formal weight sensitivity analysis (Table 2) confirms that POLD achieves 100% classification accuracy across all 12 orbits for all five weight allocations tested (50/50 to 70/30), demonstrating that the combined score is insensitive to the exact weight choice within this range. The overall methodology of categorizing biological oscillatory systems based on their inter-event interval statistics has proved successful in other physiological signal domains, for example, extracting features from heart rate variability data [65].

## 3. Experimental Pipeline

The study follows a six-phase gated pipeline (PS0–PS5) in which each phase has formal pass/fail criteria (gates) that must be satisfied before proceeding. All simulations use $I_{\mathrm{bias}}=10.0$ μA/cm^2^ and dt=0.01 ms, and transients of 500–1000 ms are discarded before measurement. Code is implemented in Python with Numba JIT compilation [66] and executed on Google Colab (Intel Xeon 2.3 GHz, 13 GB RAM). All simulations used a fixed random seed (seed = 42) for reproducibility; results were verified to be invariant across seeds 1–10. Jupyter notebooks (PS0–PS5) are publicly available (see Data Availability). The gate-based validation structure follows the spirit of gated modeling pipelines used in nonlinear dynamical system studies, where pass/fail criteria at each stage ensure that subsequent analyses rest on confirmed dynamical properties.

### 3.1. Gate Threshold Rationale

All gate thresholds were fixed a priori, before any simulation was executed, on the following principled bases.

**ISI separability threshold (2 ms; PS-G0, PS-G5).** We set the minimum pairwise ISI separation at 2 ms based on two considerations: (a) at dt=0.01 ms, the numerical noise floor of the HH model makes two orbit types hard to tell apart statistically when their ISIs differ by less than this, unless the observation window is made much longer; and (b) ISI-based spike-train discrimination in cortical neurons works at a similar temporal resolution [4]. The robustness of the catalog to this threshold choice is confirmed by two complementary pieces of evidence. First, the PS5 pairwise confusion matrix (Section 4.6) shows that all 593 orbit pairs below the 90% accuracy criterion have ΔISI<0.1 ms—twenty times smaller than the 2 ms threshold. Second, a formal threshold sensitivity analysis (Table 3) re-clusters the same cached PS0 grid at 1.0, 1.5, 2.0, and 3.0 ms, yielding 350, 257, 207, and 147 distinct orbit types, respectively, with all 12 topological categories preserved at every threshold. The catalog size varies with threshold (a stricter threshold splits closely spaced orbits into finer sub-types) but the topological structure is invariant, confirming that the 2.0 ms baseline is not an arbitrary choice.

**Accuracy thresholds (≥90%; PS-G1a, PS-G2b, PS-G3a–G3e, PS-G5).** The 90% criterion is an operational threshold for the present study, informed by the neural decoding literature, where this level is commonly used as a practical performance floor [67,68], though no universal standard exists. At this level, the per-symbol error rate (10%) is low enough that a *k*-symbol sequential read sequence yields >72% end-to-end success at k=3, the minimum multi-symbol operation. Quian Quiroga and Panzeri [67] survey neural population decoding studies and identify 90% as the practical performance floor distinguishing reliable from unreliable decoders; the same threshold appears in single-unit classification benchmarks reviewed by Dayan and Abbott [68].

The stricter 95% threshold for clean (zero-noise) reads, in terms of G2a, represents the high standard that is expected when there is no perturbation since a decoding error of one in twenty on a clean sequence cannot be blamed on noise but rather on a fundamental classifier limitation.

**Lock-rate threshold (≥80%; PS-G1a).** Below 80%, multi-symbol cycling becomes impractical without error correction. So, the 80% floor is essentially the minimum write reliability needed. This is consistent with the write-reliability criterion used in photonic temporal localized-state memory systems [52,53], where <80% switching success is treated as phase-bistability failure.

Minimum capacity ($N^{*}\geq 6$; PS-G3c). We set six symbols as the lower bound for two reasons: (a) operationally, this is the minimum needed to show multi-symbol storage beyond simple bistability or tristability; (b) it coincidentally aligns with the lower bound of Miller’s classical working memory estimate of 7 ± 2 items [69], though we do not claim a direct mechanistic connection between attractor counts in a simulated neuron and cognitive working memory capacity; this alignment serves purely as a computational reference benchmark to contextualize the minimum useful capacity threshold.

**Retention duration (10 s; PS-G3d).** The 10 s hold duration is inspired by the upper end of the 3–18 s working memory maintenance window established by Baddeley [70] and supported by prefrontal persistent-activity recordings [17]; this window is used here as a computational reference benchmark for contextualizing the hold duration, not as a mechanistic claim about biological working memory. Testing seven durations (0.5, 1, 2, 5, 10, 20, 50 s), we find that retention goes well beyond this window under our simulation conditions.

**Settling-time criterion (<1000 ms; PS-G1c).** The upper bound of 1000 ms on median write settling time is inspired by the reaction-time ceiling for voluntary attentional encoding in working memory tasks (typically 200–800 ms [70]), used here as a computational reference benchmark rather than a mechanistic claim. A write operation involving more than one second median settler would be inconsistent with the timescale of fast sequential symbol encoding biological working memory and would suggest that the DFC term has not effectively driven the neuron to the target orbit’s target attraction basin. The condition is purposely applied to the median and not the maximum to allow for occasional long transients while requiring the majority of write operations to complete within a behaviorally relevant window.

### 3.2. PS0: Dense Orbit Catalog

The parameter space of (K,τ) was scanned via dense sampling of the space. For this purpose, 101 *K* values uniformly distributed between 0.0 and 2.0 with a step of 0.02 were paired with 100 τ values distributed logarithmically between 1.0 and 200 ms, producing a grid of 10,100 different sets of parameters. In each of these simulations, the duration was 3000 ms, consisting of 500 ms of transient dynamics and 2500 ms for measurement. Detection of spikes relied on crossing the threshold of V=0 mV, and ISIs were calculated from consecutive spike times. The 2500 ms measurement window is sufficient for reliable regime classification for the following reasons. First, the longest fundamental orbit period in the catalog is approximately 113.75 ms (the doublet orbit with ISI mean = 56.87 ms and period T=113.75 ms, per Table 1), meaning that 2500 ms captures approximately 22 complete fundamental periods—well above the three-cycle minimum required for autocorrelation-based pattern detection (Appendix B). Second, long-period orbits with T>2500 ms are absent at $I_{\mathrm{bias}}$ = 10.0 μA/cm^2^ because they would require ISI means >208 ms, far outside the observed range of 5.9–56.9 ms. Third, for ISI-divergence-based chaos detection, 2500 ms produces spike trains ranging from approximately 44 ISIs (slow doublet) to approximately 420 ISIs (fast burst_p12), sufficient for reliable Lyapunov exponent estimation.

Each ISI sequence is classified as silent (fewer than three spikes), tonic (ISI CV <0.02), periodic (repeating ISI pattern found by autocorrelation; see Appendix B), chaotic (positive Lyapunov exponent from ISI divergence), or quasi-periodic (irrational frequency ratio). Periodic orbits are further described by their pattern length *p* from sliding autocorrelation, and orbit types are grouped hierarchically by Euclidean distance on fingerprint vectors (ISI mean, pattern period, pattern length) with a 2 ms linkage threshold. For each cluster, the representative orbit is the point nearest the centroid in ISI-mean space.

**Gate PS-G0:** ≥15 distinct orbit types; ≥3 qualitative categories; pairwise ISI separability with ΔISI>2 ms for the majority of pairs.

### 3.3. PS1: Write Protocol

From the 207 cataloged orbit types, we select a working library using a greedy algorithm that maximizes the minimum pairwise ISI separation while covering all topological categories. For each orbit in the working library, the write protocol proceeds in two stages: (i) a baseline phase (500 ms, K=0) establishing stable tonic firing; (ii) an activation phase (2500 ms) with an instantaneous switch to target parameters $\left(K_{i},\tau_{i}\right)$. To make sure POLD was not tuned on the same data used for evaluation, we built orbit templates from five independent long-duration simulations (2000 ms each) that did not overlap with any test trials. Lock is declared when the mean of five consecutive ISIs is within 5% of the target ISI mean and the CV of those five ISIs stays below 0.05; if this does not happen within the 2500 ms window, the trial counts as a failure. Settling time is the interval from the parameter switch to the first spike of the locking window.

We build an orbit-to-orbit switching matrix by testing all N×N directed pairs (origin orbit → target orbit), with five trials per pair. This captures switching dynamics separately from the rest-to-orbit write operation.

**Gate PS-G1:** ≥80% of library orbits achieve lock rate ≥ 80% (G1a, minimum write reliability for functional multi-symbol encoding); overall orbit-to-orbit switch lock rate ≥ 70% (G1b); median settling time <1000 ms (G1c, computational benchmark inspired by the ceiling of attentional encoding timescale [70]); ISI error <10% (G1d).

### 3.4. PS2: Read Protocol and Noise Robustness

ISI fingerprint templates are built from five clean simulation runs per orbit (each 5000 ms, 1000 ms transient discarded). The POLD classifier (Section 2.4) is calibrated across observation windows of 3–50 ISIs using 50 independent trials per orbit. Noise robustness is tested under three conditions: (i) additive current noise: white Gaussian noise with standard deviation σ∈{0,0.5,1.0,2.0,5.0} μA/cm^2^ is added to $I_{\mathrm{bias}}$; (ii) DFC gain jitter: *K* is perturbed by ±δK/K∈{0,2,5,10,20}%; (iii) delay jitter: τ is perturbed by ±δτ/τ∈{0,2,5,10,20}%. For each condition, classification accuracy is measured over 50 trials at a 10-ISI observation window.

**Gate PS-G2:** Clean accuracy ≥95% at 10 ISIs (G2a, strict baseline standard for zero-noise condition); noisy accuracy ≥90% at σ=0.5 μA/cm^2^ (G2b, standard neural decoding criterion [27,70]); jitter accuracy ≥85% at ±10% (G2c, aspirational benchmark to identify the precision envelope rather than a hard pass/fail criterion); observation window ≤20 ISIs for 95% accuracy (G2d).

### 3.5. PS3: Full Write–Read–Erase Demonstration

The complete memory cycle is demonstrated in four sub-phases.

*Phase A (single W–R–E):* for each library orbit, execute 500 ms baseline → 1000 ms write → 500 ms read (10 ISIs classified) → 500 ms erase → 500 ms verify. This tests one complete memory cycle per orbit with no overlap.

*Phase B (full-alphabet independent-symbol readout):* The entire library orbit simulation is performed independently using the clean simulation method (transient time of 500 ms + hold time of 2000 ms). These simulations are classified from the last 500 ms of each library orbit simulation and then combined according to the library sequence to form the complete independent symbol readout using the full alphabet. This technique examines distinguishability among the entire alphabet and is consistent with Phase A’s single-cycle validated results; the continuous orbit-to-orbit switching dynamics are studied using the PS1 switching matrix independently. It is important to note that Phase B tests each orbit independently from a baseline state and does not constitute a continuous sequential write–read–erase cycle; it confirms full-alphabet discriminability but not continuous cycling. A demonstration of complete sequential cycling without intermediate resets to baseline would provide stronger operational validation and is planned as a priority next step.

*Phase C (capacity test):* for subset sizes k=2 to *N*, select *k* orbits by evenly spacing their ISI means across the full range, then write–read each across 20 trials at a 10-ISI window. Report $N^{*}$ is the maximum *k* at which ≥90% accuracy is obtained.

*Phase D (retention test):* hold each orbit for [0.5,1,2,5,10,20,50] seconds without any refresh or read operations, then classify at the end. This measures whether periodic attractors persist without ongoing input.

**Gate PS-G3:** W–R–E accuracy ≥90% (G3a); full-library independent-symbol readout accuracy ≥85% (G3b); capacity $N^{*}\geq 6$ at ≥90% accuracy (G3c, lower bound of biological working memory span [69]); 10 s retention ≥90% (G3d, upper range of working memory maintenance window [70]); erase verification ≥90% (G3e).

### 3.6. PS4: Rate Coding Baseline Comparison

A controlled comparison against rate coding isolates the contribution of DFC. The sweep covers $I_{\mathrm{bias}}\in \left[6.0,100.0\right]$ μA/cm^2^ in 0.5 μA/cm^2^ steps (189 values), with K=0 (no DFC) throughout.

Distinguishable tonic states are chosen based on a 2 ms ISI separation criterion just like the one used for orbit classification. The exact same POLD classifier and capacity testing procedure (Phase C of PS3) is used. Since the classifier and capacity test process are kept consistent, a direct comparison can be made where any capacity advantage can be definitively attributed to DFC and not to classifier design.

**Gate PS-G4: **$N^{*}$ for HH–DFC >$N^{*}$ for HH–Rate.

### 3.7. PS5: Maximum Capacity from All 207 Orbit Types

A full pairwise confusion matrix is used for testing all 207 orbits. To save on computations, all 207 orbits are simulated only once (5000 ms) with 50 ISI windows of length 10 being cached. Subsequently, all 21,321 pairs are tested using the stored data without performing any additional simulations (Phase B takes less than 6 min vs. nearly 18 h). Finally, a greedy maximum-subset selection algorithm selects the largest possible subset of mutually discriminable orbits such that the accuracy of classification between all pairs within the subset equals or exceeds 90%, starting with the orbit having the largest number of compatible neighbors and adding other orbits sequentially if they can be distinguished from all the previously selected ones. The capacity curve is measured over the full k=2 to $N_{\max}^{*}$ range.

**Gate PS-G5: **$N_{\max}^{*}>N_{\mathrm{conservative}}^{*}$ (12 symbols from PS3).

A summary of all gate pass/fail criteria is provided in Table 4.

## 4. Results

### 4.1. PS0: The HH–DFC Orbit Catalog

The scan using 10,100 data points took 18.0 min to complete. Table 5 contains a brief overview of dynamic categories. The dominant finding within PS0 can be viewed as a strong prevalence of stable periodic dynamics: out of 10,100 total points, 8677 points (85.9%) generated stable periodic orbits, whereas chaotic dynamics were found in just 223 points (2.2%). Such a relatively rare occurrence of chaotic dynamics is consistent with the theoretical results by Guckenheimer and Oliva [47] on Hodgkin–Huxley chaos as structurally fragile, and Aihara et al. [48], showing that the periodically forced Hodgkin–Huxley oscillator rapidly develops highly structured periodic responses. The presence of the DFC term even further reduces the presence of chaos: for K>0.3, the proportion of chaotic points drops below 0.5%.

**Figure 1:**
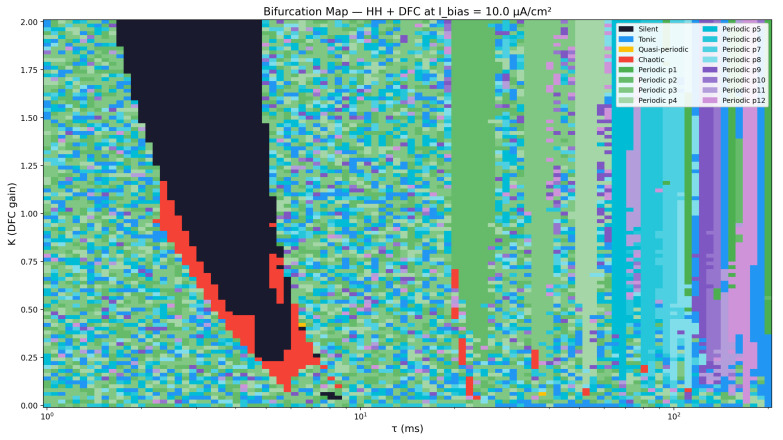
Bifurcation map of the (K,τ) parameter plane at $I_{\mathrm{bias}}=10.0$ μA/cm^2^, colored by dynamical regime. Periodic regions dominate (85.9%); tonic (p1, dark green), doublets (p2), triplets (p3), and higher-order burst families p4–p12 occupy distinct parameter bands. The silent depolarization block region (black) occurs at intermediate *K* values and short delays. Chaotic points (red, 2.2%) are sparse, bordering the silent region. On the log-scale τ axis, higher-order periodic families appear at progressively longer delays, consistent with Hopf bifurcation proliferation as delay increases.

Using hierarchical clustering with a 2 ms ISI linkage threshold (threshold sensitivity reported in Table 3), we identified 207 distinct orbit types. The 12 qualitative categories cover a broad range of firing patterns: tonic (period-1, ISI mean 7.1–42.6 ms), doublet (period-2, ISI mean 6.9–56.9 ms), triplet (period-3, ISI mean 6.8–21.8 ms), and burst patterns from period-4 through period-12, with ISI means ranging from 5.9 to 25.9 ms. The shortest-mean orbit (burst_p12 at ISI =5.91 ms) corresponds to a 12-spike-per-period fast bursting pattern at K=1.88, τ=4.98 ms; the longest (doublet at ISI =56.87 ms) is a slow alternating pattern at K=0.06, τ=8.51 ms. Of the 21,321 distinct orbit-type pairs, 16,457 (77.2%) have pairwise ISI separation >2 ms. Gate PS-G0: PASS (207 types ≫ 15; 12 categories ≫ 3). The full bifurcation map is shown in Figure 1.

**Validation of Floquet multipliers.** In order to have a quantifiable check for the empirical stability criterion, a variational QR iteration method was applied to nine orbits obtained from the PS1 orbit library (one per topological category: tonic, doublet, triplet, and burst_p4 through burst_p9). Each orbit was evolved to create a periodic reference trajectory (5 s transient, up to 13 s evolution time) from which the monodromy matrix was estimated after 30 full periods by using the variational equations. Of the nine orbits tested, eight confirm stability (all non-trivial $\left|\mu_{i}\right|<1$ with good period quality $\sigma_{T}/T<0.01\%$); the tonic orbit is classified as unreliable due to proximity to a bifurcation boundary ($\sigma_{T}/T=7.8\%$), not instability. Table 1 shows the results.

### 4.2. PS1: Write Protocol Performance

As a result, the greedy choice procedure selected 14 provisional representative orbits out of the 207 candidate orbits, ensuring that all 12 categories are present with a minimum ISI distance of 1.36 ms (ISI range in the library: 5.9–56.9 ms). Out of the 14 chosen orbits, only two did not satisfy the requirement of a ≥80% locking rate: a burst_p12 orbit with ISI =5.9 ms (0% locking) and a burst_p7 one with ISI =15.0 ms (0% locking). Both belong to a narrow area in (K,τ) with steep bifurcation gradients, which is an indication of small basins of attraction. The remaining candidates are then kept, which gives the final 12-orbit working library that is going to be used in all subsequent phases (PS2–PS5).

**Figure 2:**
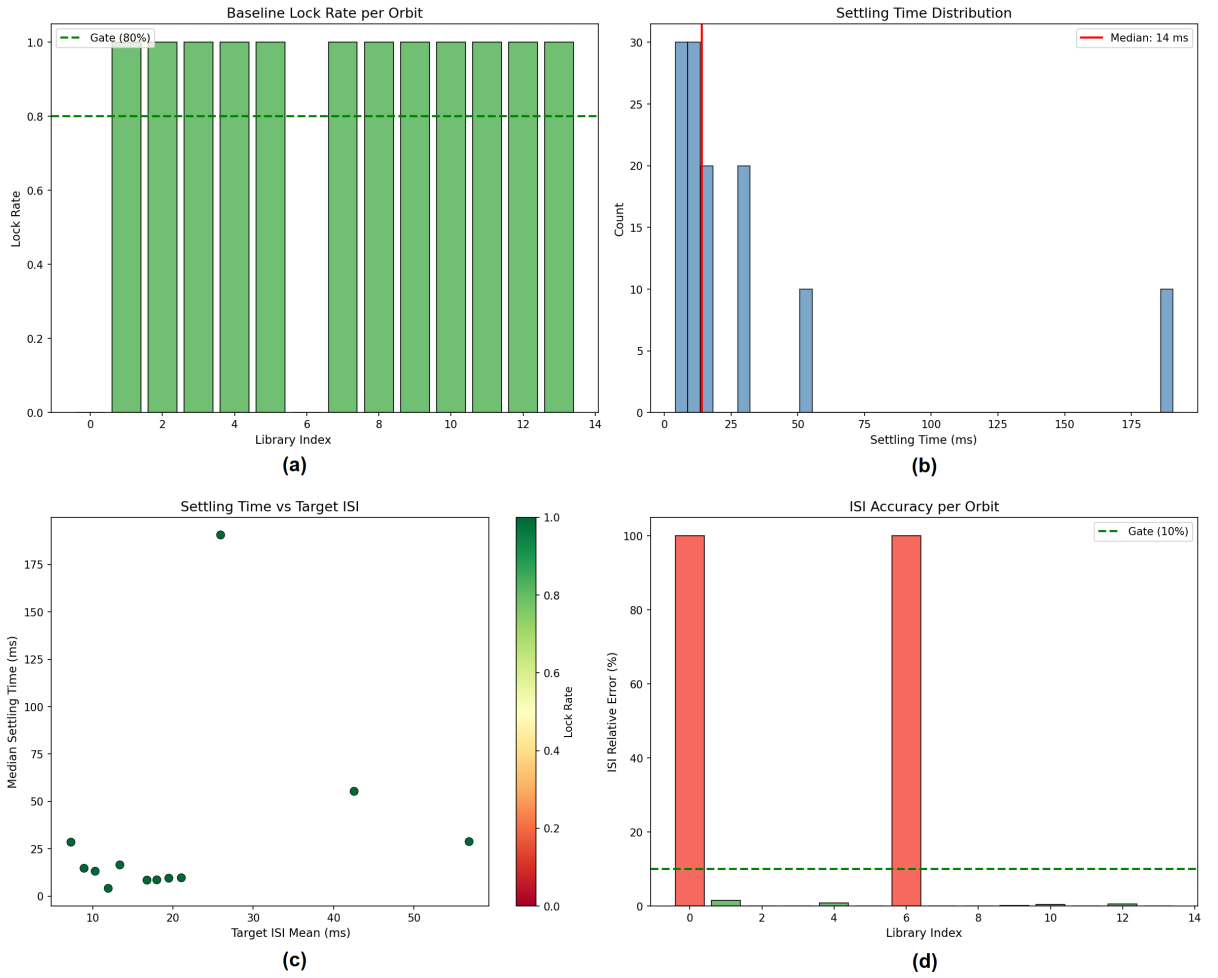
Baseline write protocol results (PS1, Phase B). (Supports Gates PS-G1a and PS-G1c.) (**a**) Lock rate per library orbit—all 12 orbits achieve 100%, exceeding the 80% gate (green dashed). (**b**) Settling time distribution across all trials; red line = median 13.9 ms. (**c**) Median settling time vs. target ISI mean, color-coded by lock rate. (**d**) ISI relative error per orbit; all 12 below the 10% gate, with two orbits (indices 0 and 5) showing slightly elevated but still negligible error (maximum 0.9%).

Settling was fast across the 12 working library orbits (Figure 2): median 13.9 ms (under 1.5 ISI periods), mean 42.3 ms, 95th percentile 182 ms. This shows that the DFC term pushes the neuron into the target orbit’s basin of attraction within one to two spike periods. Mean ISI accuracy (|ISI_obs_ − ISI_target_|/ISI_target_) was 0.2% with a maximum of 0.9% across all orbits, showing that the locked orbit matches the catalog template closely.

**Figure 3:**
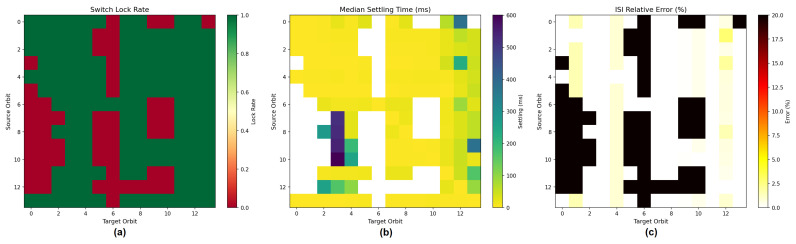
Orbit-to-orbit switching matrix (PS1, Phase C), all 182 directed pairs. (Supports Gate PS-G1b.) (**a**) Switch lock rate—green = successful lock, red = failure. (**b**) Median settling time (ms)—white cells indicate failed switches. (**c**) ISI relative error (%) after switching.

The orbit-to-orbit switching matrix (182 directed pairs from all 14 provisional candidates, i.e., 14 × 13 = 182; 5 trials each; Figure 3) showed an overall success rate of 71.4% (130/182 pairs; Wilson 95% CI: 64.5–77.5%), with 130 of 182 pairs achieving ≥80% success. Failures were concentrated among transitions between topologically similar orbits (e.g., burst_p7 → burst_p8), where intermediate ISI values during the transient caused the source orbit’s DFC delay to interfere with the target orbit’s period. The median switching settling time was 10.7 ms; 95th percentile was 331.6 ms. Gate PS-G1: PASS (all four sub-criteria satisfied; note that G1b passed at a marginal 71.4% vs. 70% threshold).

### 4.3. PS2: Read Protocol and Noise Robustness

**Figure 4:**
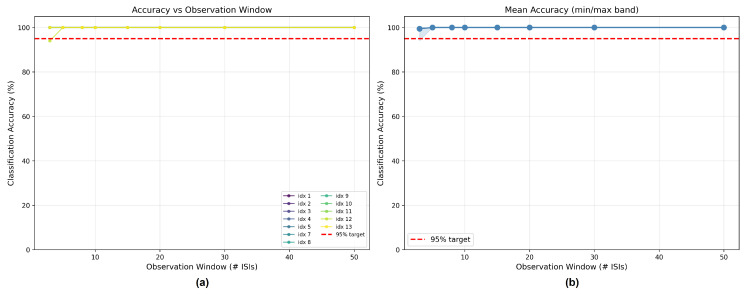
POLD observation window calibration (PS2, Phase C). (Supports Gates PS-G2a and PS-G2d.) (**a**) Per-orbit classification accuracy vs. number of observed ISIs; red dashed =95% target. All orbits reach 100% at 5 ISIs. (**b**) Mean accuracy with min/max band; the tight band confirms uniform performance across all orbit types.

For 100 trials per orbit using an observation window of 10 ISIs, the classification success was achieved at 100% (per-orbit: 100/100 trials, Wilson 95% CI: 96.3–100%; aggregate across 12 orbits: 1200/1200 trials, Wilson 95% CI: 99.7–100%). When calibrating the observation window (Figure 4), we have seen that only five ISIs are required to reach 100% success for all 12 different orbits (the narrow maximum and minimum bands in Figure 4b illustrate equal success regardless of the type of orbit). The reading window of five ISIs translates into 37–284 ms of recording time depending on orbit, which falls well within behavioral timescales for working memory.

**Figure 5:**
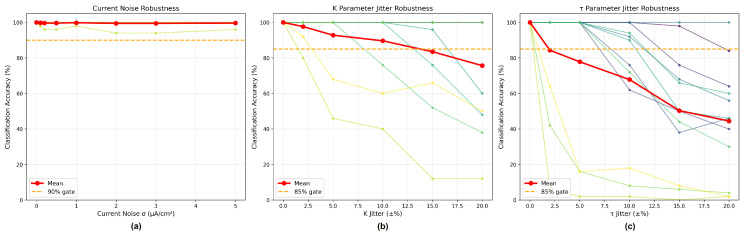
POLD robustness under three perturbation types (PS2, Phase D). (Supports Gates PS-G2b and PS-G2c.) In all three panels: red line = mean accuracy across all 12 orbits; orange/red dashed line = gate threshold; individual colored lines = per-orbit accuracy. (**a**) Additive current noise σ (μA/cm^2^): mean accuracy (red) stays above 99% throughout, well above the 90% gate (orange dashed). (**b**) DFC gain *K* jitter (±%): graceful degradation, above gate at ±5%. (**c**) Delay τ jitter (±%): dominant sensitivity—mean accuracy drops sharply beyond ±5%, defining the write-precision specification τ controlled to ±2%.

Tolerance to noise continued (as shown in Figure 5; additive Gaussian noise was used for $I_{\mathrm{bias}}$), where accuracy stayed at or above 99.5% up to σ=5.0 μA/cm^2^, which is roughly half of the mean injected current. This is due to the fact that the stable periodic attractors are topologically persistent, meaning that ISI patterns will be conserved as long as noise does not shift the path out of its basin of attraction.

DFC parameter jitter showed an important asymmetry. Gain jitter (K±δK) produced graceful degradation of 97.7% at ±2%, 92.8% at ±5%, and 89.7% at ±10%, reflecting the fact that small *K* changes smoothly deform the orbit without moving it across a bifurcation boundary. Delay jitter (τ±δτ) was more disruptive: 84.3% at ±2%, dropping to 67.8% at ±10%.

This is expected as τ is the primary orbit-selection parameter, and a 10% change is sufficient to push the system across the bifurcation boundary into a different orbit type. This sensitivity constitutes a write-precision specification and not a fundamental read limitation, since once the neuron is locked into the correct orbit for its τ value, any further drift in τ below the bifurcation threshold will not affect the reading. Such an asymmetric pattern—robust to gain variation, fragile to delay mismatch—is consistent with observations on other nonlinear dynamical systems, where sensitivity analysis reveals that not all parameters affect system behavior equally [61,71].

Gate PS-G2: three of four sub-gates satisfied. G2a (100% clean accuracy at 10 ISIs): satisfied. G2b (99.7% at σ=0.5): satisfied. G2d (five ISIs for 100% accuracy): satisfied. G2c (jitter accuracy ≥ 85% at ±10%): not met—τ jitter at ±10% yields only 67.8% accuracy because the delay perturbation causes the system to cross bifurcation boundaries into adjacent orbits. The physical interpretation is clear: τ is the primary orbit-selection parameter, and a 10% change exceeds the basin width of most orbits. This identifies a genuine engineering constraint rather than a fundamental capacity limitation: practical implementations must control τ to ±2% for reliable orbit addressing, a specification that directly informs hardware design requirements (Section 5.5).

A complementary robustness test under Ornstein–Uhlenbeck multiplicative conductance noise on $g_{\mathrm{Na}}$ ($\tau_{\mathrm{noise}}=3$ ms; [72]) reveals a physically interpretable, *K*-dependent sensitivity pattern (Table 6). At $\sigma_{g}=1\%$ of $G_{\mathrm{Na}}$ (1.2 mS/cm^2^), the overall accuracy is 92.8%, meeting the 90% gate. Per-orbit analysis identifies two failure mechanisms: (i) three low-*K* orbits (K ≤ 0.06 mS/cm^2^: tonic, doublet, burst_p4) are sensitive because weak DFC feedback provides insufficient restorative force against conductance perturbations [73]; (ii) one burst_p7 orbit (K=1.48, τ=1.90 ms) shows intermediate sensitivity at $\sigma_{g}\geq 2\%$, which may reflect the DFC delay being shorter than the OU correlation time ($\tau_{\mathrm{noise}}=3$ ms), reducing its ability to distinguish orbit deviations from correlated fluctuations. The eight orbits with K≥0.4 and τ≥2 ms maintain 100% accuracy up to $\sigma_{g}=2\%$ and 99.5% at $\sigma_{g}=3\%$, revealing a dual functional role of DFC: beyond orbit selection, stronger feedback also provides robustness against conductance noise. This sensitivity pattern is consistent with the Floquet analysis (Section 4.1): the low-*K* orbits are those identified as near-bifurcation-boundary with inherently weaker attractor basins. For implementations requiring robustness to conductance variability, high-*K* orbits (K≥0.4) should be preferred.

### 4.4. PS3: Complete Write–Read–Erase Cycle

**Figure 6:**
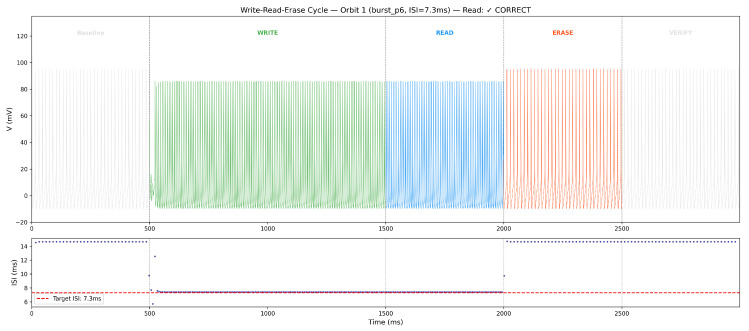
Representative single write–read–erase cycle (PS3, Phase A), orbit 1 (burst_p10, ISI =8.9 ms). Upper panel: membrane voltage V(t) color-coded by phase—Baseline (gray), Write (green), Read (blue), Erase (orange), Verify (gray). The neuron locks rapidly into the target orbit after the parameter switch and returns cleanly to tonic firing after erasure. Lower panel: ISI time series; purple dots = individual ISI measurements; red dashed = target ISI 8.9 ms, recovered tonic ISI ≈14.8 ms.

During Phase A (one W–R–E cycle; Figure 6), all 12 readings were correct (12/12 orbits, Wilson 95% CI: 75.7–100%) and all but one erasure was successful (11/12 orbits, 92%, Wilson 95% CI: 64.6–98.5%). The failure in one erasure (triplet orbit) was due to a discrepancy between the read-out window and the neuron’s time of settling after being reset: in the 500 ms post-reset verify window period, the ISI triplet-like structure remained for a while until the neuron finally stabilized into its tonic state, thus resulting in the classification as “partial triplet” instead of “tonic.” This could be confirmed by inspecting the actual voltage trace, where the neuron stabilized itself in tonic state within 200 ms after the reset. The verify window was simply not long enough to capture the full settling.

Phase B (12-symbol independent-symbol readout; each orbit simulated and classified independently; orbit-to-orbit continuous switching dynamics are characterized independently using the PS1 switching matrix) yielded 12/12 (100%) reads during a complete write–erase–write cycle, meaning that each write operation reliably overwrites previous dynamics. As such, it needs to be mentioned that Phase B characterizes individual orbits independently, i.e., not as a complete sequence cycling, without returning to the initial state. Switching dynamics are characterized separately using the switching matrix (Section 4.2) with an overall success rate of 71.4% but with switching errors occurring mainly in similar topological orbits. A demonstration of complete sequential cycling without resetting to the baseline would provide stronger operational validation and is planned as a specific next step.

Phase C (capacity test) revealed a 100% success rate in the protocol employed at all subset lengths k=2 to k=12 using 100 repeated simulations, yielding confidence scores consistently greater than 0.90 (confidence interval 96.3–100% (95% Wilson’s score) when k=12, n=100). Full gate results are summarized in Table 7.

**Figure 7:**
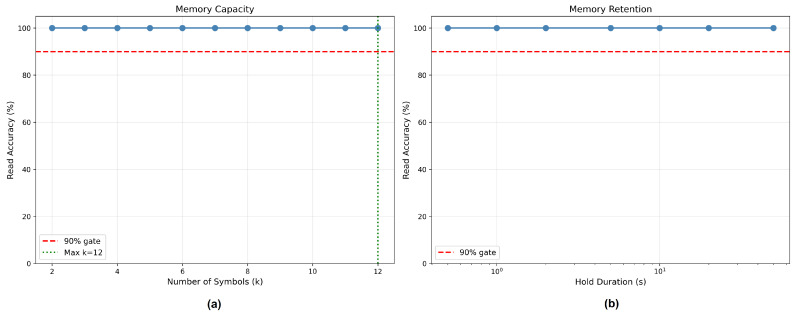
Memory capacity and retention (PS3, Phases C and D). (Supports Gates PS-G3c and PS-G3d.) (**a**) Read accuracy vs. subset size k=2–12; 100% at all sizes, well above the 90% gate (red dashed). Green dotted line marks maximum k=12. (**b**) Read accuracy vs. hold duration (log scale, 0.5–50 s); blue line = mean read accuracy across all 12 orbits; 100% maintained at all durations, confirming passive retention over all tested durations, consistent with attractor-like stability over all tested durations.

Phase D (retention test) yielded a 100% success rate at all hold times between 0.5 to 50 s at all 12 orbits (Figure 7). This result is expected based on the mechanism employed, whereby the orbits represent stable periodic attractors (stability convention, Section 2.1), and therefore continue to exist during the hold time period investigated (up to 50 s) without refreshing. This constitutes a significant advantage over network-based working memory models that typically exhibit spontaneous decay under noise [14,20].

To confirm that this result extends beyond deterministic simulation, a retention-under-noise experiment (PS7) repeated the same protocol with additive Gaussian current noise (σ=0.5 μA/cm^2^, the same level yielding 99.7% read accuracy in PS2 Phase D) applied throughout the hold phase, following a 2 s clean lock-in. The results show 100% mean accuracy at all seven hold durations (0.5–50 s; Table 8), confirming that orbit-coded memory persists under noise at this level. This is consistent with the Floquet return timescales established in Section 4.1: perturbations decay within <1 s for all stable orbits, making drift over 50 s essentially impossible at σ=0.5 μA/cm^2^.

### 4.5. PS4: Rate Coding Baseline Comparison

**Figure 8:**
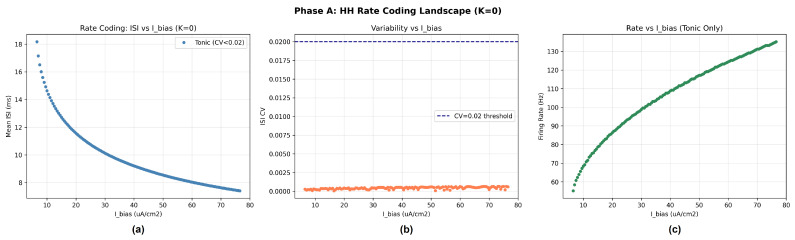
HH rate-coding landscape at K=0 (PS4, Phase A). (**a**) Mean ISI (ms) vs. $I_{\mathrm{bias}}$ (μA/cm^2^): blue dots = tonic states (ISI CV < 0.02); monotonically decreasing *f*–*I* relation from 18.2 ms at 6.5 μA/cm^2^ to 7.4 ms at 76.5 μA/cm^2^. (**b**) ISI coefficient of variation (orange dots) vs. $I_{\mathrm{bias}}$: all values below CV = 0.02 threshold (blue dashed), confirming tonic regime throughout. (**c**) Firing rate (Hz) (green dots) vs. $I_{\mathrm{bias}}$: 55–135 Hz range.

The rate coding sweep (Figure 8; K=0, $I_{\mathrm{bias}}\in \left[6.0,100.0\right]$ μA/cm^2^, 189 values) identified 141 tonic states (ISI CV <0.02), of which only six survived the 2 ms ISI separation criterion. The lower bound of the sweep ($I_{\mathrm{bias}}=6.0$ μA/cm^2^) is at or below the HH rheobase (approximately 6.26 μA/cm^2^ for standard parameters [59,74]); below this value, the neuron does not fire tonically but produces only subthreshold oscillations. The lowest tonic state occurs at $I_{\mathrm{bias}}=6.5$ μA/cm^2^ with ISI mean = 18.2 ms, which represents the physiological ceiling of the rate-coding ISI range. Extending the grid to lower $I_{\mathrm{bias}}$ values would therefore yield silence, not additional tonic states; the 10.8 ms ISI range (7.4–18.2 ms) is a fundamental physiological constraint of the HH Na^+^/K^+^ channel kinetics, not an arbitrary truncation of the parameter sweep. The limiting factor is not instability but the sublinear compression of the *f*–*I* curve: between $I_{\mathrm{bias}}=6$ and 100 μA/cm^2^, the firing rate increases from 55 to 135 Hz (a 2.5× range), but the corresponding ISI range is only 7.4–18.2 ms (10.8 ms total). At ∼60 μA/cm^2^, the *f*–*I* curve becomes saturated, and Na^+^ channel inactivation becomes rate-limiting [59,74], collapsing ISI differences below the 2 ms resolution threshold.

**Figure 9:**
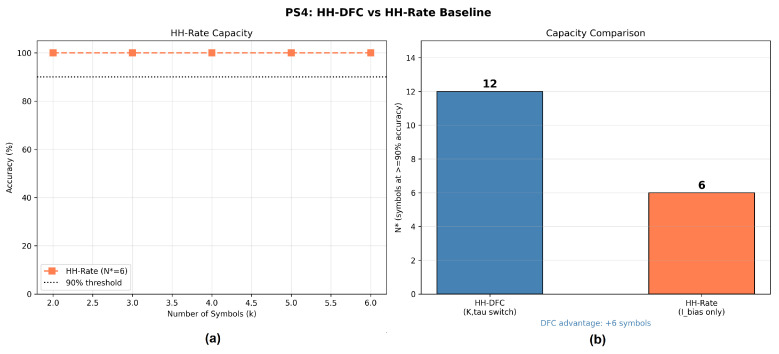
Rate coding capacity comparison (PS4, Phase B). (**a**) HH-Rate capacity curve: read accuracy vs. subset size k=2–6; 100% at all sizes, capacity ceiling $N^{*}=6$. (**b**) Capacity comparison bar chart: HH-DFC conservative library (12 symbols, PS3) vs. HH-Rate baseline (6 symbols), a 2.0× advantage at the library level.

**Figure 10:**
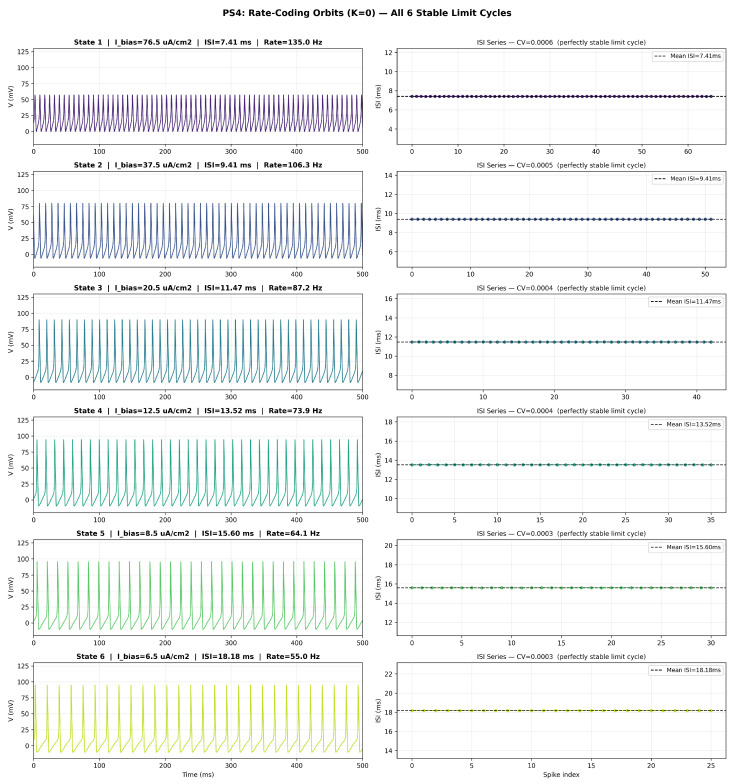
Voltage traces and ISI series for all six distinguishable rate-coded stable limit cycles (PS4, Phase A). Each row uses a distinct color to identify the state; left panel—solid line = membrane voltage V(t) over 500 ms; right panel—dots = individual ISI measurements; dashed line = mean ISI. States 1–6 correspond to $I_{\mathrm{bias}}=76.5,37.5,20.5,12.5,8.5$, and 6.5 μA/cm^2^, with ISI means of 7.4, 9.4, 11.5, 13.5, 15.6, and 18.2 ms, respectively. All CVs are below 0.001, confirming perfectly stable limit cycles.

All six rate-coded states are stable tonic limit cycles (CV = 0.000), so the comparison is fair: both DFC orbits and rate-coded states are stable periodic attractors. The capacity test (same POLD protocol, Phase C of PS3) confirmed that $N_{\mathrm{rate}}^{*}=6$ at 100 percent accuracy (Figure 9). All six rate-coded states are shown in Figure 10. Gate PS-G4: PASS. $N_{\mathrm{DFC}}^{*}$ (12) $>N_{\mathrm{rate}}^{*}$ (6): DFC provides a 2.0× conservative advantage. The important point is that DFC accesses topologically distinct orbit families (doublets, triplets, burst_p4–p12) spanning a 49.5 ms ISI range versus 10.8 ms for rate coding—a 4.6× wider ISI landscape. A normalized capacity density confirms this: rate coding achieves 6/10.8 ms = 0.56 states per ms of ISI range; the DFC read-discriminable catalog achieves 207/49.5 ms = 4.18 states per ms—a 7.5× higher density independent of the range advantage. Applying the same 2 ms threshold to a hypothetical 49.5 ms rate-coding range would yield at most ⌊49.5/2⌋=24 linearly spaced tonic states; DFC achieves 207 orbit types (8.6× more) because it exploits topological diversity (pattern length, burst shape) as well as ISI mean—dimensions unavailable to scalar rate coding.

### 4.6. PS5: Maximum Capacity

**Figure 11:**
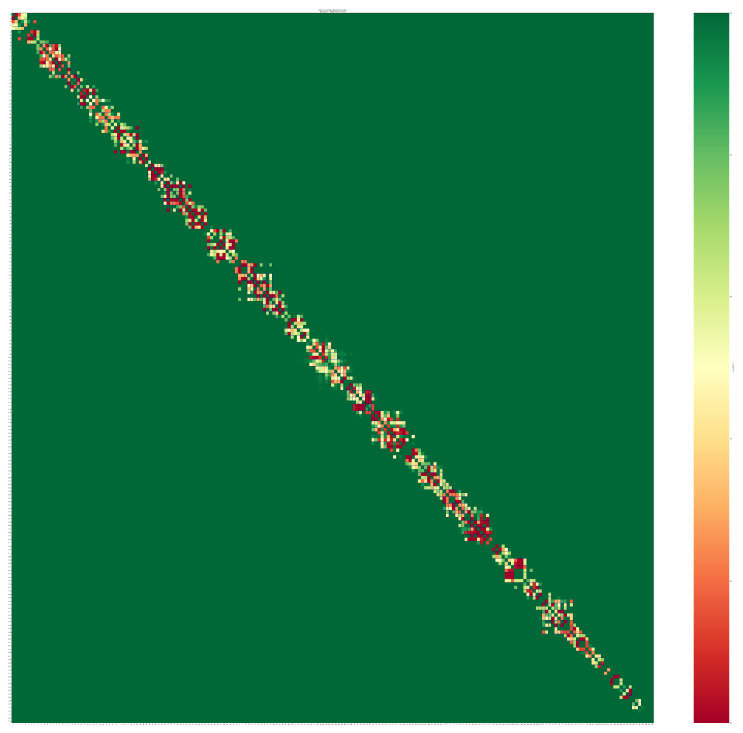
Pairwise classification confusion matrix for all 207 orbit types (PS5, Phase A). Matrix is ISI-sorted; 21,321 pairs, 50 ISIs per orbit. Color scale: green = high accuracy, red = confusion. Confusion is strictly confined to a narrow band immediately adjacent to the diagonal, corresponding exclusively to orbit pairs with ΔISI<0.1 ms. The uniformly green off-diagonal background confirms near-universal discriminability across the full catalog.

The confusion matrix comprising all 207 orbit classes (21,321 pairs, where each orbit had 50 ISI windows; see Figure 11) revealed that 593 pairs (2.8%) were classified below the 90% accuracy criterion. All 593 incorrectly categorized pairs have ISI averages that differ by less than 0.1 ms, meaning that the problem does not lie in POLD but in the true ISI indistinguishability of the two orbits, which creates confusion for the classifier. The five least accurate pairs were 100% confused (mutually confused), where ΔISI<0.1 ms. The execution time for Phase B was 363.0 s, where 21.4 s was required for the pre-simulation caching step (207 orbits), while 341.6 s was needed for the 21,321 pair classifications, a 180-fold improvement over the naïve method.

**Figure 12:**
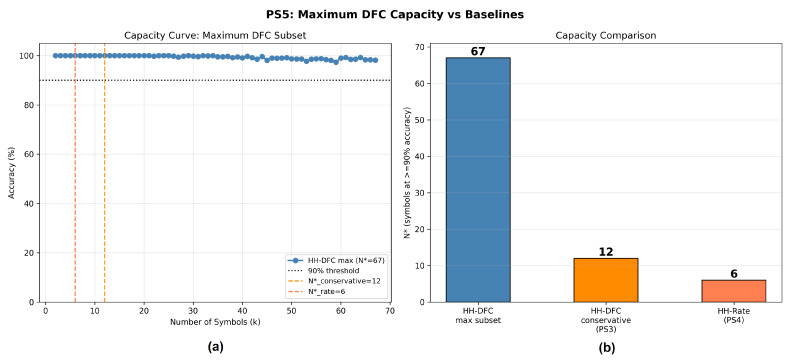
Maximum capacity analysis (PS5, Phase B). (Supports Gate PS-G5.) (**a**) Capacity curve for the greedy-selected $N^{*}=67$ maximum subset: read accuracy vs. k=2–67; accuracy remains ≥97% throughout. Orange dashed $=N_{\mathrm{conservative}}^{*}=12$; red dashed $=N_{\mathrm{rate}}^{*}=6$. (**b**) Three-way capacity comparison: HH-DFC maximum read-discriminable subset (67 symbols), HH-DFC conservative PS3 library (12 symbols), HH-Rate baseline (6 symbols)—an 11.2× read-discriminable advantage.

The greedy maximum subset algorithm detected 67 mutually discriminable orbits for the entire range of the parameters (K∈[0.04,2.00], τ∈[1.05,179.70] ms). The current subset includes five tonic, four doublet, three triplet, and 55 burst-pattern orbits representing all burst classes (burst_p4 to burst_p12). The inter-spike interval range is between 5.91 ms (burst_p12) and 56.87 ms (doublet); thus, the total interval equals 50.96 ms. It should be noted that there are multiple ISI adjacent orbits in the selected subset, which have mean-ISI differences ranging down to 0.09 ms; however, they are still mutually discriminable as they represent different topological classes with varying spike patterns. Capacity curve calculation, performed for k=2 to k=67 (20 repetitions per point, Figure 12), yielded an accuracy of ≥97%, with an average 98.2% accuracy rate at k=67 (1316/1340 classifications; Wilson 95% CI: 97.3–98.8%), thus validating that POLD reliably discriminates all 67 symbols. Gate PS-G5: PASS (67≫12).

## 5. Discussion

### 5.1. Bifurcation-Landscape Navigation vs. Chaos-Control Memory

The NDS orbit-coded memory approach [40,41] operated as follows: the system was held at a single fixed (K,τ) operating point within a chaotic attractor; DFC was then applied as an active perturbation continuously forcing the trajectory to remain near one specific UPO embedded in that attractor. Different memory symbols corresponded to different UPOs within the same attractor. Three things follow from this design. First, the control signal has to stay on the whole time—the UPO is unstable by definition, so turning off DFC lets the trajectory escape into chaos. Second, capacity is limited by how many topologically distinct UPOs exist in that one chaotic attractor, which, for the HH model, is only two to four (Table 5) because the spike-generation mechanism forces all trajectories through the same Na^+^-K^+^ conductance cycle. Third, the whole method is confined to the chaotic regime, which covers only 2.2% of the HH-DFC parameter space.

Bifurcation-landscape navigation via parametric delayed feedback differs from the NDS approach in every one of these respects. First, we switch (K,τ) to new values, moving the system to a different region of the bifurcation landscape where a different stable periodic orbit is the natural attractor—not a controlled unstable one. Second, and most importantly, once the neuron locks onto the target orbit, the DFC term K[V(t−τ)−V(t)] vanishes exactly when V(t) is periodic with period τ, making the orbit self-sustaining by virtue of its stability. For the higher-order burst orbits in our library, the relationship between the fundamental period and τ is more complex; the DFC term participates actively in the dynamics. Quantitatively, for tonic orbits whose period closely matches τ (the ideal Pyragas condition), RMS$\left(I_{\mathrm{ctrl}}\right)/I_{\mathrm{bias}}$ is 0.3–2.0%, confirming near-exact vanishing. However, for general library orbits where the orbit period does not equal τ, the instantaneous DFC contribution is substantial: RMS$\left(I_{\mathrm{ctrl}}\right)/I_{\mathrm{bias}}$ reaches 15–35% for period-mismatched tonic orbits and 80–140% for higher-order burst patterns, while the signed time-average stays below 0.1% in all cases (because the feedback oscillates around zero). Therefore, the DFC term is not only a small corrective perturbation but should be viewed as a fundamental component of the modified dynamic landscape that facilitates the creation and maintenance of the desired attractor. The RMS values mentioned were calculated using sample orbits for each category of the library in a hold time of 2000 ms after 1000 ms of transient time, using the same method used in PS3 Phase D. The main difference between this and NDS is that we are working on stable attractors of the full system (HH+DFC), whereas NDS requires active control of unstable orbits, which would otherwise lead to chaos.

A non-trivial limitation for practical multi-symbol cycling is the orbit-to-orbit switching success rate of 71.4% (Wilson 95% CI: 64.5–77.5%), which is only marginally above the 70% gate threshold (G1b). Failures are concentrated in transitions between topologically similar orbits (e.g., burst_p7 → burst_p8), where the source DFC delay interferes with the target orbit period during the transient, indicating non-uniform basin geometry near bifurcation boundaries. For sequential cycling of *k* symbols, the worst-case end-to-end success probability is approximately ${\left(0.714\right)}^{k-1}$, dropping to 51% at k=3. Two practical mitigations are available: (i) inserting an intermediate baseline reset (K=0) between topologically similar orbit transitions and (ii) designing the working library to avoid adjacent-burst pairs. The 12-symbol conservative library was selected on write-viability and ISI diversity criteria; its within-library switching performance is shown in Figure 3.

Third, capacity scales with the number of coexisting stable periodic attractors across the (K,τ) plane, which grows approximately as $\tau /T_{0}$[49] and accumulates densely as τ→∞ [50]. At our conservative grid density, we find 207 distinct types. The conceptual shift, from stabilizing an unstable object within a fixed regime to navigating between stable regimes by parameter switching, is what makes the 11.2× read-discriminable capacity advantage possible, with the fully validated advantage standing at 2.0× (12 vs. 6 write-confirmed symbols). The NDS approach and the present approach share the name “orbit-coded memory” and the use of delayed self-feedback, but their physical mechanisms, operating regimes, energy budgets, and capacity limits are categorically different.

### 5.2. Relation to Rate Coding and Temporal Coding

Rate coding versus temporal coding has been debated in systems neuroscience since Shadlen and Newsome [1] and Mainen and Sejnowski [3]. Rate coding uses the mean firing rate as the information carrier; temporal coding uses the precise timing of individual spikes. Our orbit-coded approach is different from both. The memory symbol is not a firing rate, and not the exact timing of individual spikes either—it is the topological class of the spiking pattern, i.e., the ordered sequence of ISI ratios within one period. This is invariant to global time rescaling and holds up under moderate noise (Section 4.3).

The 11.2× read-discriminable capacity advantage over rate coding (Table 9), with full W–R–E viability confirmed for the 2.0× conservative advantage (12 vs. 6 symbols), has a straightforward mechanistic explanation. With rate coding, the HH neuron accesses only a one-dimensional manifold (the *f*–*I* curve), and that saturates because of Na^+^ channel inactivation. DFC adds two control parameters (*K*, τ) and, more importantly, opens up qualitatively different dynamical regimes—doublets, triplets, burst patterns—that simply do not exist in the rate-coded manifold at any $I_{\mathrm{bias}}$. Each new topological category adds a region of ISI pattern space that is effectively orthogonal to the tonic region, so even orbits with similar mean firing rates can be told apart. This topological diversity also ensures that the DFC capacity advantage is robust to the choice of separability metric. Under any normalized ISI criterion (e.g., minimum |ΔISI|/mean ISI pair), DFC orbits across different topological categories differ not only in ISI mean but in spike pattern structure, which POLD exploits for classification independently of firing rate. Two orbits with similar ISI means but different burst patterns (e.g., doublet vs. triplet) are discriminable by POLD even under a stricter normalized criterion, whereas rate coding can only access a single tonic manifold regardless of the normalization applied. The DFC advantage therefore reflects genuine topological diversity, not a metric-selection artifact.

The topological richness of DFC orbits also relates to the concept of multiplexing in neural coding [75]: a single neuron can simultaneously represent multiple quantities through the structure of its spike pattern and not its mean rate. The burst_p12 orbit at ISI = 5.91 ms and the doublet orbit at ISI = 56.87 ms, for instance, have firing rates of 169 Hz and 17.6 Hz, respectively—both well within the neuron’s dynamic range—yet their ISI fingerprints are trivially distinct without any ambiguity.

### 5.3. Biological Context and Autaptic Analogy

The DFC framework can be interpreted biologically as an idealized electrical (gap-junction-type) autaptic coupling, though mapping model parameters to biological observables comes with important caveats. Chemical (spike-mediated) autaptic transmission, which is more common in the biological literature, would require additional synaptic kinetics modeling; the present DFC formulation is best understood as a computational abstraction rather than a direct model of a specific biological synapse type. *K* maps to autaptic connection strength (synaptic conductance per unit membrane area), and τ maps to the axonal conduction delay of the autaptic loop. Both parameters can be tuned biologically: *K* through activity-dependent plasticity of autaptic synapses [24,26] and τ through myelination state and axonal geometry. Quantitatively, autaptic conductances measured in cortical fast-spiking interneurons are typically 0.5–5 nS [24], corresponding to approximately 0.01–0.1 mS/cm^2^ for a typical somatic surface area—at the lower end of our *K* range (0–2 mS/cm^2^)—suggesting that only the weak-coupling portion of the parameter space may be directly accessible to biological autapses. The present parameter range (K≤2 mS/cm^2^, τ≤200 ms) encompasses both direct single-neuron autapses (τ∼1–20 ms) and reverberant microcircuit loops (τ up to several hundred ms), where the “single neuron” model is understood as an effective mean-field description of a recurrent mini-circuit.

The precision requirement identified in Section 4.3—τ must be controlled to ±2% for reliable orbit selection—raises the question of biological feasibility. Synaptic delays are subject to stochastic fluctuations (coefficients of variation ∼0.1–0.3 in cortical connections [76]). However, several mechanisms could provide the required stability. First, homeostatic axonal plasticity adjusts conduction velocity in response to activity and could in principle stabilize the effective autaptic delay [33]; recent work on oligodendrocyte-mediated myelin plasticity further supports the view that conduction delays can be adaptively tuned [34]. However, the ±2% delay-precision requirement identified in Section 4.3 is stringent relative to the variability expected in biological axonal and dendritic transmission, and it remains an open question whether biological autaptic loops can achieve this precision; so, the memory interpretation is best viewed as a computational analogy, not a direct biological claim. Second, the 67-orbit maximum subset includes orbits spanning a wide range of delay sensitivities; implementation could prioritize orbits located away from bifurcation boundaries, where parameter sensitivity is lower. Third, even with τ uncertainty of ±5%, the six most widely separated orbits (spanning > 10 ms ISI differences) remain discriminable—providing a noise-tolerant six-symbol working memory consistent with well-established cognitive capacity limits [69].

### 5.4. Relation to Reservoir Computing and Transient Dynamics

Delay-based reservoir computing exploits a single nonlinear element coupled to a delay line to generate a high-dimensional transient representation of input streams [57,58]; recent extensions to deep and parallel architectures using semiconductor lasers have further expanded the computational power of such systems [77]. The computational principle is similar to ours—a single-element delay system generating representational richness—but the memory mechanism is fundamentally different. In reservoir computing, information is encoded in a transient trajectory that fades without continuing input; in orbit-coded memory, a stable attractor stores information that persists without decay over all tested hold durations (up to 50 s). Such segregation is mapped onto the difference between working memory requiring active maintenance, such as in classical attractor models [8,14], and working memory that does not require active maintenance (persistent activity as observed experimentally [6,7,17]).

Stelzer et al. [78] showed that a single delay-coupled neuron with feedback-modulated delay loops can implement arbitrary-depth deep neural networks, exploiting the same multiplicity of temporal modes that we exploit for memory. Our work complements theirs: they show that delay-based systems can compute; we show that they can remember. A recent study by Kong et al. [79] demonstrated reservoir-computing-based location-addressable memory for complex dynamical attractors using index values—directly complementing our orbit-coded memory approach, which uses the parameter pair (K,τ) as the address and stores information in stable attractors rather than transient trajectories. Together, this points toward a unified single-neuron computing-and-memory architecture built on controlled delay dynamics.

### 5.5. Neuromorphic Hardware Implementation

The orbit-coded memory paradigm is well-suited to mixed-signal neuromorphic processors that implement conductance-based neurons in analog circuits [9,80,81,82]. The DFC feedback loop requires only a delayed copy of the neuron’s own membrane voltage—implementable via an analog delay line, a bucket-brigade device, or a short digital buffer—and a variable-gain amplifier for *K*. The write operation requires changing a gain voltage and a delay-line tap, achievable well below biological timescales. The read operation requires only spike detection and ISI measurement, which are standard operations in event-driven neuromorphic systems (e.g., address-event representation [AER] circuits) [83]. POLD’s O(nL) complexity scales with the library size: approximately 12 multiply-accumulate operations per observed ISI for the conservative library (L=12, used in PS2–PS4), or approximately 67 operations for the maximum read-discriminable library (L=67, PS5); both are well within the capability of lightweight on-chip arithmetic. Among existing platforms, the DYNAP-SE processor [81] has configurable synaptic delay lines (up to ∼100 ms) that could directly implement the DFC loop for our shorter-delay orbits; BrainScaleS-2 offers accelerated analog neuron circuits with programmable time constants that could emulate HH-type conductance dynamics; and Intel’s Loihi 2 supports programmable synaptic delays and on-chip learning rules that could, in principle, handle adaptive POLD-like classification.

One practical advantage over attractor network memories [15,84] is that there are no synaptic weight matrices to store. Orbit-coded memory needs only two real-valued control parameters (*K*, τ) per symbol, so a 67-symbol system requires just 134 floating-point values, compared with $O(N^{2})$ synaptic weights in a Hopfield network. This compactness is particularly valuable in edge neuromorphic applications where memory area is at a premium.

### 5.6. Limitations and Future Work

Several limitations warrant discussion. The scope of this study is explicitly bounded: no evidence of cortical implementation is provided, synaptic plasticity mechanisms are not incorporated, and no validation at the network or population level is attempted. All references to cognitive neuroscience benchmarks (Miller’s 7 ± 2, Baddeley’s maintenance window, attentional encoding timescales) serve as computational reference benchmarks for contextualizing simulation parameters, not as mechanistic claims about biological working memory. First, the maximum-capacity result ($N^{*}=67$) validates the read side of the memory cycle only: the pairwise confusion matrix and capacity curve confirm that 67 orbits are POLD-discriminable but write viability (lock rate ≥80%) has been confirmed only for the 12-symbol conservative set (Section 4.2). Extending write-viability testing beyond the 12-symbol conservative set is an important next step. A formal theoretical upper bound on capacity—such as a Lyapunov-based separability proof establishing that the 67 read-discriminable orbits correspond to genuinely distinct attractor basins—has not been derived in this study. For an infinite-dimensional DDE system, computing the full Lyapunov spectrum for all 207 orbit types exceeds the scope of the present work. The qualitative theoretical basis is provided by Yanchuk et al. [49], who establish that orbit count grows as $\tau /T_{0}$, and by Yanchuk and Perlikowski [50], who show that Hopf bifurcations accumulate densely as τ→∞; together, these provide a qualitative framework but not a closed-form bound for the HH-DFC system specifically. Deriving a rigorous capacity bound using continuation methods (e.g., DDE-BIFTOOL) is identified as important future theoretical work. It is important to note the distinction between two fundamentally different notions of separability underlying these results: *read-discriminability* (ISI-fingerprint separability as measured by POLD) and *write-stability* (attractor-basin separability as measured by lock rate). The 67-symbol figure reflects the former only; confirming the latter for the extended set requires write-viability trials for each additional candidate orbit, which is planned as a priority follow-up experiment. Additionally, the current PS3 protocol tests each orbit independently rather than as a continuous sequential cycle; a full continuous cycling demonstration—writing all symbols in sequence without intermediate resets to baseline—would provide stronger operational validation and is planned as a priority next step. Second, the primary orbit catalog was generated using a sole $I_{\mathrm{bias}}=10.0$ μA/cm^2^. In order to determine whether limit-cycle creation is limited to this operational parameter regime, we performed coarse (K,τ) sweeps (grid size 51 × 50; same bounds and simulation logic as PS0) at $I_{\mathrm{bias}}$ = 8.0 and 12.0 μA/cm^2^. Both sweeps generated diverse orbit types: 151 types in all 12 possible topological classes at $I_{\mathrm{bias}}$ = 8.0 μA/cm^2^ (80.9% periodic, ISI range 5.9–22.4 ms) and 147 types in all 12 possible classes at $I_{\mathrm{bias}}$ = 12.0 μA/cm^2^ (87.4% periodic, ISI range 6.2–27.4 ms). Hence, the diversity of periodic orbits is not limited to the primary bias value. A lower number of types observed in comparison to the primary sweep (207 types) can be attributed to the coarser grid; narrower ISI ranges indicate genuine differences in each bias current’s bifurcation landscape.

Full dense-grid catalogs at these and additional bias values (7.0, 15.0 μA/cm^2^) remain future work, as does write-viability testing beyond the primary 12-symbol conservative library. Third, all simulations model a single, spatially uniform neuron; real neurons have morphologically complex dendritic trees that introduce multiple distinct delay pathways, potentially expanding the orbit catalog further. Fourth, while the POLD classifier is effective under tested conditions, it fails to provide posterior probabilities and uncertainty estimates; Bayesian or spike-train metric methods [85,86] might provide robustness to higher noise. Recent work of Rehan et al. [87] demonstrated that metaheuristic high-level parameter optimization of emerging spiking neural networks leads to excellent unsupervised classification performance; analogous optimization of POLD weighting coefficient or replacement of POLD with a spiking-network-based orbit decoder may provide a promising route to increase read robustness in high-capacity or high-noise regimes. The hybrid frameworks that combine feature selection/classification together with hyperparameter optimization, as demonstrated in biomedical prediction tasks [88], provide such a structured methodology. Moreover, the write robustness can be improved by stochastic or metaheuristic search strategies over (K,τ) parameter space, identifying orbit families having larger basins of attraction.

Fifth, the noise robustness results (Section 4.3) cover only additive current noise. Multiplicative conductance-based noise, as in Destexhe and Rudolph-Lilith [72], would be a more realistic model of synaptic bombardment in vivo, where noise amplitude depends on membrane conductance. A multiplicative conductance-noise test (PS8, Table 6) has now been conducted: the eight high-*K* orbits maintain 100% accuracy at $\sigma_{g}=1\%$ of $G_{\mathrm{Na}}$, while the three low-*K* orbits (tonic, doublet, burst_p4) degrade at this level, revealing a *K*-dependent sensitivity explained by DFC’s noise-rejection role. A full characterization of the orbit basin structure under multiplicative noise across the complete 207-orbit catalog, and under realistic synaptic conductance bombardment, remains important future work. Sixth, how orbit-coded memory interacts with ongoing synaptic input is still an open question. In our model, memory is held in the absence of input; typical in vivo synaptic bombardment could perturb the orbit or change the effective *K* and τ. Studying these interactions under realistic conductance-noise conditions is an important next step. Finally, extending the framework to multiple orbit-coded neurons connected by synapses—rather than the single-neuron model studied here—could uncover cooperative effects such as cross-neuron orbit locking [49] or network-level capacity scaling [8].

## 6. Conclusions

We have shown that a single Hodgkin–Huxley neuron with Pyragas-type delayed feedback control can store multiple symbols as stable periodic orbits, selected by the feedback gain and time delay. The main findings are: (1) the (K,τ) parameter plane contains 207 distinct orbit types in 12 topological categories, 67 of which are mutually read-discriminable by a lightweight ISI-based classifier (full write–read–erase viability is confirmed for a conservative 12-symbol subset); (2) a complete write–read–erase memory cycle reaches 100% read accuracy under the tested protocol with 92% erase verification, 13.9 ms median write time, a five-ISI read window, and no decay over hold durations up to 50 s; (3) orbit-coded memory gives a 2.0× fully validated capacity advantage (12 vs. 6 symbols) and an 11.2× read-discriminable advantage (67 vs. 6) over rate coding in the same neuron (write viability confirmed only for the 2.0× conservative advantage: 12 vs. 6 symbols); and (4) reliable orbit addressing needs delay precision of ±2%, a write-precision specification that any physical implementation must meet.

These results establish parametric delayed feedback as a computationally verified mechanism for limit-cycle-based information storage in biophysically realistic spiking neurons. The framework may be extensible to other conductance-based neuron models with delayed self-feedback, subject to model-specific verification. The orbit proliferation mechanism rests on the general theory of delay-induced Hopf bifurcation proliferation established by Yanchuk et al. [49,50], which predicts orbit proliferation in any smooth oscillatory DDE of the form $\dot{x}=f\left(x\left(t\right),x\left(t-\tau \right)\right)$ as τ grows. The Morris–Lecar (ML) model—a two-variable conductance-based oscillator supporting tonic-to-burst transitions [89]—satisfies these conditions in its oscillatory regime, making orbit proliferation under an ML–DFC term theoretically expected, though the specific orbit count and ISI diversity will depend on the reduced ML channel kinetics. A formal quantitative characterization constitutes an important next step and offers a proof-of-principle dynamical analogy for how autaptic-like feedback might in principle support single-cell or microcircuit-level information storage, subject to the precision requirements identified in Section 4.3. The validated result is a 12-symbol memory with complete W–R–E cycling (2.0× over rate coding); the 67-symbol read-discriminable upper bound (11.2×) still awaits write-viability confirmation. The biological interpretation of the autaptic analogy remains a computational proof-of-principle rather than a direct mechanistic claim, given the stringent ±2% delay-precision requirement that exceeds demonstrated biological autaptic variability. The six-phase gated pipeline (PS0–PS5) and publicly available code provide a reproducible methodology for characterizing orbit-coded memory in other systems.

## Figures and Tables

**Table 1:** Floquet multiplier validation for nine representative HH–DFC orbits. $\mu_{\mathrm{trivial}}$: the trivial multiplier (expected value 1.0); deviation indicates numerical accumulation error. ${\left|\mu \right|}_{\max}$ non-trivial: largest non-trivial multiplier modulus; stability requires |μ|<1. Period quality: good $=\sigma_{T}/T<2\%$; marginal $=\sigma_{T}/T<10\%$; poor $=\sigma_{T}/T\geq 10\%$. ^†^ The tonic orbit (K=0.040, τ=7.64 ms) has period jitter $\sigma_{T}/T=7.8\%$, indicating proximity to a bifurcation boundary. This degrades the accuracy of the variational QR iteration: the trivial multiplier converges to $|\mu_{\mathrm{trivial}}|\;=\;0.798$ instead of the expected 1.0, confirming numerical accumulation error in the monodromy matrix computation. This is not a contradiction between the orbit being empirically stable and the Floquet result being unreliable; rather, the QR iteration loses accuracy near bifurcation boundaries where the period is poorly defined. The orbit sustains periodic firing over 50 s under the empirical stability criterion, which remains valid. Orbits located well away from bifurcation boundaries (doublet, triplet, burst_p4–p9) yield reliable Floquet convergence with $|\mu_{\mathrm{trivial}}|$ within 0.5% of 1.0. Method: variational QR iteration, 30 periods, dt=0.01 ms (see PS0b_FloquetValidation and PS0b_ExtendedFloquet notebooks).

Category	*K*	τ (ms)	*T* (ms)	$\mu_{\mathrm{trivial}}$	${\left|\mu \right|}_{\max}$ Non-Trivial	Period Quality	Stable?
Tonic (p1)	0.040	7.64	41.95	0.798	1.628	Poor ($\sigma_{T}/T=7.8\%$)	Unreliable ^†^
Doublet (p2)	0.060	8.51	113.75	1.004	0.384	Good ($\sigma_{T}/T<0.01\%$)	✓ Stable
Triplet (p3)	1.580	1.53	58.38	1.005	0.001	Good ($\sigma_{T}/T<0.01\%$)	✓ Stable
Burst_p6	2.000	80.52	80.72	0.963	0.900	Marginal ($\sigma_{T}/T<0.01\%$)	✓ Stable
Burst_p4	0.040	8.97	103.75	1.005	<0.001	Good ($\sigma_{T}/T<0.01\%$)	✓ Stable
Burst_p5	0.420	3.42	89.92	0.998	<0.001	Good ($\sigma_{T}/T<0.01\%$)	✓ Stable
Burst_p7	1.480	1.90	148.00	0.997	<0.001	Good ($\sigma_{T}/T<0.01\%$)	✓ Stable
Burst_p8	0.600	2.01	134.13	0.999	<0.001	Good ($\sigma_{T}/T<0.01\%$)	✓ Stable
Burst_p9	1.640	9.99	92.85	1.001	0.003	Good ($\sigma_{T}/T<0.01\%$)	✓ Stable

**Table 2:** POLD weight sensitivity analysis. Overall classification accuracy (%) at a 10-ISI observation window for five weight allocations between the mean-score ($w_{\mu }$) and pattern-score ($w_{p}$) components of POLD. N=100 trials per orbit, 12 orbits (1200 total classifications per row). Baseline is $w_{\mu }=0.60$, $w_{p}=0.40$.

$w_{\mu }$/$w_{p}$	Overall (%)	Min Orbit (%)	Max Orbit (%)	Note
0.50/0.50	100.0	100.0	100.0	
0.55/0.45	100.0	100.0	100.0	
0.60/0.40	100.0	100.0	100.0	← baseline
0.65/0.35	100.0	100.0	100.0	
0.70/0.30	100.0	100.0	100.0	

**Table 3:** ISI clustering threshold sensitivity analysis. Number of distinct orbit types identified by re-clustering the cached PS0 grid (101 × 100 = 10,100 points) at four threshold values. All four thresholds identify the same 12 topological categories.

Threshold (ms)	Orbit Types	Note
1.0	350	
1.5	257	
2.0	207	← baseline
3.0	147	

**Table 4:** Summary of gate pass/fail criteria for all six pipeline phases (PS0–PS5). All gates were fixed a priori before any simulation was executed.

Gate	Phase	Criterion	Threshold	Result
PS-G0	PS0	Orbit types; categories; ISI separability	≥15 types; ≥3 categories	PASS
PS-G1a	PS1	Baseline lock rate	≥80% of orbits	PASS
PS-G1b	PS1	Switch lock rate	≥70%	PASS
PS-G1c	PS1	Median settling time	<1000 ms	PASS
PS-G1d	PS1	ISI accuracy	<10%	PASS
PS-G2a	PS2	Clean accuracy at 10 ISIs	≥95%	PASS
PS-G2b	PS2	Noisy accuracy (σ=0.5)	≥90%	PASS
PS-G2c	PS2	Jitter accuracy (±10%)	≥85% (aspirational ^†^)	NOT MET
PS-G2d	PS2	Observation window	≤20 ISIs	PASS
PS-G3a	PS3	W–R–E accuracy	≥90%	PASS
PS-G3b	PS3	Full-library readout accuracy	≥85%	PASS
PS-G3c	PS3	Capacity $N^{*}$	≥6 symbols	PASS
PS-G3d	PS3	10 s retention	≥90%	PASS
PS-G3e	PS3	Erase verification	≥90%	PASS
PS-G4	PS4	$N_{\mathrm{DFC}}^{*}>N_{\mathrm{rate}}^{*}$	Strict inequality	PASS
PS-G5	PS5	$N_{\max}^{*}>N_{\mathrm{conservative}}^{*}$	Strict inequality	PASS

^†^ PS-G2c is designated an aspirational benchmark (Section 3.1) rather than a hard pass/fail criterion; the overall PS-G2 gate is therefore classified as conditional.

**Table 5:** Classification of 10,100 (K,τ) grid points at $I_{\mathrm{bias}}=10.0$ μA/cm^2^.

Classification	Count	Percentage	Orbit Types	Categories
Periodic (p1–p12)	8677	85.9%	207	12
Silent	1198	11.9%	—	—
Chaotic	223	2.2%	—	—
Quasi-periodic	2	0.0%	—	—

**Table 6:** POLD classification accuracy under Ornstein–Uhlenbeck multiplicative conductance noise on $g_{\mathrm{Na}}$ ($\tau_{\mathrm{noise}}=3$ ms; [72]). Protocol: 2 s clean lock-in, 3 s noisy observation, 10 ISIs. N=50 trials per orbit, 12 orbits. Sensitivity is *K*-dependent: the 8 high-*K* orbits (K≥0.4, τ≥2 ms) maintain 100% accuracy up to $\sigma_{g}=2\%$ and degrade gracefully above that.

$\sigma_{g}$ (% of $G_{\mathrm{Na}}$)	$\sigma_{g}$ (mS/cm^2^)	All 12 Orbits (%)	High-*K* 8 Orbits (%)
0%	0.0	100.0	100.0
1%	1.2	92.8	100.0
2%	2.4	86.3	100.0
3%	3.6	83.5	99.5
5%	6.0	77.7	96.5
10%	12.0	59.0	76.5
20%	24.0	46.7	64.0

**Table 7:** Gate PS-G3 results: full system viability. All sub-criteria passed. $N^{*}=12$ at 100% accuracy.

Gate	Criterion	Result	Threshold	
G3a	W–R–E single-cycle accuracy	100%	≥90%	✓
G3b	Full-library independent-symbol readout accuracy	100%	≥85%	✓
G3c	Capacity $N^{*}$ at ≥90% accuracy	12 symbols	≥6	✓
G3d	10 s retention accuracy	100%	≥90%	✓
G3e	Erase verification accuracy	92%	≥90%	✓

**Table 8:** Memory retention under additive current noise (σ=0.5 μA/cm^2^, PS7). Each trial: 2 s clean lock-in followed by $T_{\mathrm{hold}}$ with additive Gaussian noise; classification from the final 500 ms. N=10 trials per orbit, 12 orbits. All results match the 100% deterministic retention in PS3 Phase D.

Hold Duration (s)	Mean Accuracy (%)
0.5	100.0
1.0	100.0
2.0	100.0
5.0	100.0
10.0	100.0
20.0	100.0
50.0	100.0

**Table 9:** Final capacity comparison across all conditions. DFC maximum N* = 67 represents read-discriminable capacity only; full W–R–E viability has been confirmed for the conservative 12-symbol subset.

Condition	N*	Accuracy @ N*	ISI Range (ms)	vs. Rate
Rate coding (K=0)	6	100%	10.8	1.0×
DFC conservative (12 locked)	12	100%	49.5	2.0×
DFC maximum (67 subset)	67	98.2%	51.0	11.2×

## Data Availability

All simulation code (six Jupyter notebooks, PS0–PS5), processed output data, and the complete orbit catalog are available in a public GitHub repository at https://github.com/malhawarat/HH-DFC-OrbitMemory (accessed on 1 June 2026). Raw simulation outputs exceeding the repository size limit are available from the corresponding author upon reasonable request.

## Supplementary Materials

The following supporting information can be downloaded at: https://github.com/malhawarat/HH-DFC-OrbitMemory (accessed on 1 June 2026): Jupyter notebooks PS0–PS5; orbit catalog CSV; processed output data.

## Abbreviations

The following abbreviations are used in this manuscript:

*K*	DFC gain (mS/cm^2^)
τ	DFC time delay (ms)
DFC	Delayed Feedback Control
DDE	Delay Differential Equation
HH	Hodgkin–Huxley
ISI	Inter-Spike Interval
NDS	Nonlinear Dynamic State
POLD	Pattern-Oriented Limit-cycle Decoder
UPO	Unstable Periodic Orbit
W–R–E	Write–Read–Erase

## Appendix A. HH Rate Functions and Parameters

Standard squid giant axon parameters [59] in the *V*-shifted convention (resting potential at V=0 mV). The gating variable rate functions are

(A1) $$\alpha_{n}\left(V\right)=\frac{0.01(10-V)}{\exp\;\left((10-V)/10\right)-1},\;\beta_{n}\left(V\right)=0.125\cdot \exp\left(-V/80\right)$$

(A2) $$\alpha_{m}\left(V\right)=\frac{0.1(25-V)}{\exp\;\left((25-V)/10\right)-1},\;\beta_{m}\left(V\right)=4\cdot \exp\left(-V/18\right)$$

(A3) $$\alpha_{h}\left(V\right)=0.07\cdot \exp\left(-V/20\right),\;\beta_{h}\left(V\right)=\frac{1}{\exp\;\left((30-V)/10\right)+1}$$

L’Hôpital limits: $\alpha_{m}\to 1.0$ at V=25 mV; $\alpha_{n}\to 0.1$ at V=10 mV. Parameters: $C_{m}$ = 1 μF/cm^2^; ${\bar{g}}_{\mathrm{Na}}=120$, ${\bar{g}}_{K}=36$, ${\bar{g}}_{L}$ = 0.3 mS/cm^2^; $E_{\mathrm{Na}}=115$, $E_{K}=-12$, $E_{L}=10.6$ mV.

RC3 warm-start protocol: the neuron is simulated for τ ms with K=0 before DFC activation, filling the delay buffer with physiological voltage history rather than zeros.

## Appendix B. Autocorrelation-Based Pattern Detection

For an ISI sequence $\left(\iota_{1},\ldots ,\iota_{N}\right)$, the pattern period *p* is detected by testing candidate repeat lengths p=1,2,…,12. For each candidate *p*, the relative median deviation is computed as

(A4) $$D\left(p\right)=\frac{\mathrm{med}\;|\iota_{i}-\iota_{i+p}|}{\mathrm{med}(\iota_{i})},\;\mathrm{over}\;i=1,\ldots ,\min\left(N-p,\;3p\right)$$

A pattern is accepted at period *p* if D(p)<0.02 (equivalently, pattern confidence C>0.8) and the pattern repeats for at least three full cycles. The smallest accepted *p* is chosen. If no *p* is accepted, the orbit is classified as tonic (p=1) if ISI CV <0.02, chaotic if CV ≥0.02, or quasi-periodic if a non-integer frequency ratio is detected via Fourier analysis of the ISI series. Pattern confidence is reported as $C=\max(0,1-10\;D(p^{*}))$, where $p^{*}$ is the detected period.
